# Aldolase B-driven lactagenesis and CEACAM6 activation promote cell renewal and chemoresistance in colorectal cancer through the Warburg effect

**DOI:** 10.1038/s41419-023-06187-z

**Published:** 2023-10-10

**Authors:** Yu-De Chu, Li-Chun Cheng, Siew-Na Lim, Ming-Wei Lai, Chau-Ting Yeh, Wey-Ran Lin

**Affiliations:** 1 Liver Research Center, Chang Gung Memorial Hospital, Taoyuan, 333 Taiwan; 2 Department of Neurology, Linkou Chang Gung Memorial Hospital, Taoyuan, 333 Taiwan; 3 Division of Pediatric Gastroenterology Department of Pediatrics, Linkou Chang Gung Memorial Hospital, Taoyuan, 333 Taiwan; 4 Molecular Medicine Research Center, Chang Gung University, Taoyuan, 333 Taiwan; 5 College of Medicine, Chang Gung University, Taoyuan, 333 Taiwan; 6 Department of Hepatology and Gastroenterology, Linkou Chang Gung Memorial Hospital, Taoyuan, 333 Taiwan

**Keywords:** Colorectal cancer, Metabolomics, Cancer metabolism

## Abstract

Colorectal cancer (CRC) is a prevalent malignancy worldwide and is associated with a high mortality rate. Changes in bioenergy metabolism, such as the Warburg effect, are often observed in CRC. Aldolase B (ALDOB) has been identified as a potential regulator of these changes, but its exact role in CRC cell behavior and bioenergetic homeostasis is not fully understood. To investigate this, two cohorts of CRC patients were analyzed independently. The results showed that higher ALDOB expression was linked to unfavorable prognosis, increased circulating carcinoembryonic antigen (CEA) levels, and altered bioenergetics in CRC. Further analysis using cell-based assays demonstrated that ALDOB promoted cell proliferation, chemoresistance, and increased expression of CEA in CRC cells. The activation of pyruvate dehydrogenase kinase-1 (PDK1) by ALDOB-induced lactagenesis and secretion, which in turn mediated the effects on CEA expression. Secreted lactate was found to enhance lactate dehydrogenase B (LDHB) expression in adjacent cells and to be a crucial modulator of ALDOB-mediated phenotypes. Additionally, the effect of ALDOB on CEA expression was downstream of the bioenergetic changes mediated by secreted lactate. The study also identified CEA cell adhesion molecule-6 (CEACAM6) as a downstream effector of ALDOB that controlled CRC cell proliferation and chemoresistance. Notably, CEACAM6 activation was shown to enhance protein stability through lysine lactylation, downstream of ALDOB-mediated lactagenesis. The ALDOB/PDK1/lactate/CEACAM6 axis plays an essential role in CRC cell behavior and bioenergetic homeostasis, providing new insights into the involvement of CEACAM6 in CRC and the Warburg effect. These findings may lead to the development of new treatment strategies for CRC patients.

## Introduction

Colorectal cancer (CRC) is a prevalent malignancy with high mortality worldwide, including Taiwan. According to a report by Global Cancer Observatory, about one million new cases were diagnosed in 2020, with 5.7 billion deaths due to CRC complications [1]. Despite advances in diagnosis, many patients are diagnosed with intermediate or late stages, making surgical resection the most effective curative method of treatment. However, surgery is often not feasible for late-stage tumors, and recurrence or metastasis is frequently observed within five years of surgical resection. To address these challenges, combination therapies may be employed alone or in conjunction with surgery to shrink tumors and prevent relapse or metastasis in early-stage patients [2].

The development of CRC is initiated by the transformation of premalignant adenomatous polyps (APs) through the adenoma-to-carcinoma sequence, which is induced by genetic mutations, inflammatory responses, growth factor expression, and activation/inactivation of cancer-associated signaling pathways [3, 4]. Metabolic reprogramming in the tumor microenvironment (TME) has emerged as a critical factor that can regulate tumorigenesis and/or progression and identify potential biomarkers and targets for anticancer therapy in several cancer types, including CRC [5–12].

Bioenergy is derived from anaerobic lactic acid fermentation, aerobic glycolysis, and mitochondrial oxidative phosphorylation (OXPHOS) [13]. Cancer cells secrete more lactate than normal cells, a phenomenon referred to as the “Warburg effect,” even under aerobic conditions. This is observed in many cancer types, including CRC, and is primarily driven by lactate dehydrogenase (LDH) composed of two subunits, LDHA and LDHB, and monocarboxylate transporters (MCTs). LDHA is proposed as a key LDH subunit for converting pyruvate to lactate following PDK1-4 activation, whereas LDHB plays a crucial role in lactate oxidation and generating pyruvate for OXPHOS utilization, promoting tumor growth and progression [14–18].

Previous studies suggest that Aldolase B (ALDOB) expression is associated with changes in AP or CRC bioenergetics [7]. Elevated ALDOB levels correlate with unfavorable postoperative prognosis in CRC patients, colon cancer liver metastases, and response to neoadjuvant chemoradiotherapy in rectal cancer patients [19–21]. Existing evidence also suggests that ALDOB may promote epithelial-mesenchymal transition, increase the risk of liver metastasis, and mediate fructose metabolism to potentiate cell proliferation [19, 20]. However, the association of ALDOB with cell proliferation and chemosensitivity in terms of bioenergetic changes remains unclear. Therefore, the purpose of this study is to investigate the role of ALDOB in modulating CRC cell behavior and bioenergetic homeostasis and their potential interactions.

## Results

### Association of ALDOB levels with CRC prognosis: elevated expression in tumor tissue and correlation with poor outcomes in patients

**Fig. 1:**
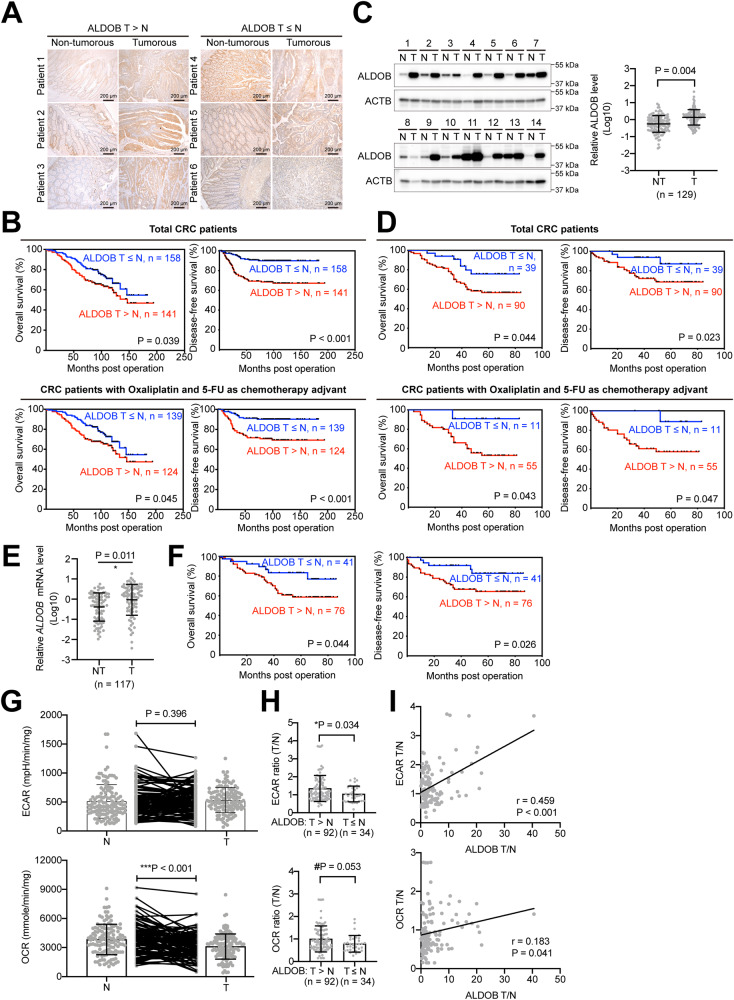
Upregulation of ALDOB in CRC is associated with unfavorable postoperative prognosis and bioenergetic changes. **A** Representative IHC sections used to score ALDOB intensity in CRC in nontumor or tumor areas. **B** Kaplan–Meier analysis of prognosis in CRC patients, overall (*n* = 299, upper panel) or treated with chemotherapy (*n* = 263, lower panel), stratified from the ratio of ADLOB expression intensities in nontumor and tumor areas derived from (**A**). **C** Representative Western blots of ALDOB in both nontumor and tumor tissues. Signal intensities obtained from Western blots were statistically summarized in the right panel. **D** Kaplan–Meier analysis of prognosis in CRC patients, overall (*n* = 129, upper panel) or treated with chemotherapy (*n* = 66, lower panel), stratified by the ratio of ADLOB expression levels in nontumor and tumor. **E** Statistical analysis of relative *ALDOB* mRNA levels in nontumor and tumor tissues (*n* = 117) from the real-time quantitative polymerase chain reaction. **F** Kaplan–Meier analysis of prognosis in CRC patients (*n* = 117), stratified by the ratio of *ADLOB* mRNA levels in nontumor and tumor. **G** Extracellular acidification rate (ECAR) and oxygen consumption rate (OCR) obtained using the Seahorse assay. **H** Statistical analysis of bioenergetic changes in CRC patients with high or low tumor ALDOB expression. **I** Pearson’s analysis of the correlation between ALDOB expression and bioenergetic changes in CRC.

In order to investigate the potential role of ALDOB in CRC, the levels of ALDOB were assessed in patient tissues using immunohistochemical (IHC) staining. The staining intensities in both nontumor and tumor areas on a single slide were compared. Patients were then grouped based on their level of ALDOB in the tumor area, with one group having higher levels than the nontumor area (*T* > *N*), and the remaining patients having lower or equivalent levels (*T* ≤ *N*), as illustrated in Fig. 1A. Comparison of baseline characteristics between these two subgroups, as shown in Table 1, revealed that patients with higher levels of ALDOB in tumorous tissue exhibited a higher degree of local invasion (*P* = 0.048) and circulating carcinoembryonic antigen (CEA) (*P* = 0.048).

**Table 1:** Baseline data for patients with CRC included in this study.

Variable	ALDOB *T* > *N* (*n* = 141)	ALDOB *T* ≤ *N* (*n* = 158)	*P*
Gender, male, *n* (%)	84 (59.6%)	81 (51.3%)	0.149
Age, years, mean ± SD	54.6 ± 11.0	56.6 ± 11.6	0.067
Height, cm, mean ± SD	161.3 ± 7.8	161.6 ± 8.0	0.945
Weight, kg, mean ± SD	62.5 ± 10.3	64.3 ± 12.8	0.417
BMI, kg/m^2^, mean ± SD	24.0 ± 3.2	24.5 ± 3.9	0.227
Tumor location, left sided, *n* (%)	91 (64.5%)	97 (61.4%)	0.574
Tumor differentiation, poor, *n* (%)	18 (12.8%)	22 (13.9%)	0.769
Local invasion, pT3 + pT4, *n* (%)	138 (97.9%)	147 (93.0%)	**0.048**
Maximum tumor size, cm, mean ± SD	18.9 ± 15.9	19.4 ± 16.8	0.870
CEA, ng/mL, mean ± SD	9.5 ± 23.0	7.8 ± 23.8	**0.048**
Adjuvant chemotherapy, *n* (%)	132 (93.6%)	152 (96.2%)	0.307
Distance to serosa, mm, mean ± SD	2.5 ± 4.2	3.9 ± 10.4	0.597

*BMI* body mass index, *CEA* carcinoembryonic antigen.

Bold values indicate statistical significance *P* < 0.05.

Cox regression analysis was performed to investigate the association of clinicopathological factors and ALDOB levels with the prognosis of CRC patients. As presented in Table 2, only ALDOB expression levels (*P* = 0.033) and receipt of adjuvant chemotherapy (*P* = 0.002) were found to be linked with overall survival (OS). Multivariate analysis indicated that both ALDOB expression and patient experience with adjuvant chemotherapy were independent predictors of OS (*P* = 0.033 and 0.026, respectively). Similarly, ALDOB expression levels (*P* < 0.001), gender (*P* = 0.022), and adjuvant chemotherapy experience (*P* = 0.009) were significantly associated with disease-free survival (DFS). The multivariate analysis revealed that ALDOB expression, gender, and patient experience with adjuvant chemotherapy were independent factors for DFS (*P* < 0.001, 0.033, and 0.026, respectively).

**Table 2:** Cox proportional hazard analysis for association between clinical factors and OS or DFS.

Clinical parameters	Univariate analysis		Multivariate analysis	
	HR (95% CI)	*P*	HR (95% CI)	*P*
*For OS*				
ALDOB level, high expression = 1	1.536 (1.036–2.278)	**0.033**	1.496 (1.008–2.220)	**0.046**
Gender, male = 1	1.186 (0.797–1.765)	0.399		
Age, per year increase	0.993 (0.976–1.011)	0.445		
Height, per cm increase	1.009 (0.984–1.035)	0.492		
Weight, per kg increase	0.998 (0.981–1.015)	0.812		
BMI, per kg/m^2^ increase	0.977 (0.922–1.034)	0.423		
Tumor location, left sided = 1	1.080 (0.718–1.623)	0.713		
Differentiation, well+moderate = 1	0.701 (0.411–1.197)	0.193		
Local invasion, per grade increase	1.237 (0.854–1.793)	0.261		
Tumor size, per cm increase	0.990 (0.975–1.004)	0.160		
CEA, per ng/mL increase	1.001 (0.994–1.008)	0.803		
Adjuvant chemotherapy, yes = 1	0.342 (0.172–0.680)	**0.002**	0.359 (0.180–0.715)	**0.004**
Distant to serosa, per mm increase	0.984 (0.951–1.018)	0.362		
*For DFS*				
ALDOB level, high expression = 1	3.745 (2.083–6.733)	**<0.001**	3.502 (1.943–6.312)	**<0.001**
Gender, male=1	1.913 (1.099–3.329)	**0.022**	1.828 (1.049–3.187)	**0.033**
Age, per year increase	0.987 (0.965–1.009)	0.232		
Height, per cm increase	1.020 (0.988–1.053)	0.223		
Weight, per kg increase	1.003 (0.982–1.025)	0.783		
BMI, per kg/m^2^ increase	0.986 (0.917–1.060)	0.703		
Tumor location, left sided = 1	1.225 (0.710–2.116)	0.466		
Differentiation, well+moderate = 1	1.425 (0.722–2.814)	0.307		
Local invasion, per grade increase	1.452 (0.899–2.347)	0.128		
Tumor size, per cm increase	0.989 (0.971–1.007)	0.232		
CEA, per ng/mL increase	1.005 (0.997–1.012)	0.217		
Adjuvant chemotherapy, yes = 1	0.323 (0.139–0.754)	**0.009**	0.380 (0.162–0.892)	**0.026**
Distant to serosa, per mm increase	0.992 (0.954–1.030)	0.664		

*HR* hazard ratio, *BMI* body mass index, *CEA* carcinoembryonic antigen.

Bold values indicate statistical significance *P* < 0.05.

The role of ALDOB in CRC prognosis was further confirmed by Kaplan–Meier analysis. Patients with low ALDOB expression in tumor tissue (*T* ≤ *N*) were found to have a more favorable prognosis (Fig. 1B upper panel). As approximately 80% of patients had received 5-fluorouracil (5-FU) and oxaliplatin combination therapy, additional analysis was performed to include these patients. Again, good prognosis was observed in patients with low ALDOB expression in tumor tissue (*T* ≤ *N*) (Fig. 1B lower panel), suggesting that ALDOB expression sensitizes CRC to the anticancer agents 5-FU and oxaliplatin.

To validate these findings, an additional 129 paired patient frozen tissues were collected for western blot analysis. It was observed that ALDOB expression was significantly elevated in tumor tissue (*P* = 0.004) (Fig. 1C). Using the ratio of ALDOB levels in tumor and nontumor tissues, Kaplan–Meier analysis further confirmed that patients with high ALDOB expression in tumor tissue (*T* > *N*) had an unfavorable prognosis (Fig. 1D upper panel). Sixty-six out of 129 patients who received 5-FU and oxaliplatin-related adjuvant chemotherapy were included for further analysis on ALDOB expression and therapeutic efficacy. As demonstrated in Fig. 1D lower panel, patients with higher ALDOB levels in their tumor tissue exhibited poorer therapeutic outcomes, suggesting that ALDOB expression affects the sensitivity of CRC cells to 5-FU or oxaliplatin.

ALDOB protein expression has been implicated previously and in this study as a potential prognostic biomarker [19–21] (Fig. 1A–D). However, it is unclear whether ALDOB mRNA levels can also serve as a predictor. Among the 129 patients analyzed in Fig. 1C, ALDOB mRNA expression levels were evaluated in 117 cases. As depicted in Fig. 1E, ALDOB mRNA was found to be elevated in tumor tissue (*P* = 0.011). Corresponding to the protein level findings, patients with high ALDOB mRNA levels in tumor tissue (*T* > *N*) had an unfavorable prognosis (Fig. 1F). These results suggest that tissue ALDOB mRNA levels can also be used as a valuable prognostic biomarker in CRC patients.

### Association of ALDOB expression with OXPHOS and aerobic glycolytic activity in CRC, but not in AP

In previous studies on CRC tissues, a significant reduction in OXPHOS activity, expressed as oxidative consumption rate (OCR), was observed, while the aerobic glycolytic capacity, represented as extracellular acidification rate (ECAR), was only slightly altered [7]. A subsequent study using tissue from a subset of patients also confirmed these findings, as depicted in Fig. 1G. However, when patient subgroups were stratified based on the ratio of ALDOB levels in tumor and nontumor tissues, those with high tumor ALDOB expression (*T* > *N*) showed significantly increased ECAR (*P* = 0.034) and a borderline increase in OCR (*P* = 0.053) (Fig. 1H). Further analysis revealed a strong positive correlation between ALDOB expression in tumors and ECAR ratios (*r* = 0.459, *P* < 0.001) and a weaker positive correlation with OCR ratios (*r* = 0.183, *P* = 0.041) (Fig. 1I).

In AP, ECAR was markedly increased, whereas OCR showed no significant difference compared to normal mucosa [7]. This observation was supported by a confirmatory study (Fig. S1A). Interestingly, in contrast to CRC, higher ALDOB expression in AP (*P* > *N*) was associated with lower ECAR and OCR (Fig. S1B). Additionally, a weak negative correlation was found between ALDOB expression and ECAR (*r* = −0.252, *P* = 0.045) (Fig. S1C).

### Impact of increased ALDOB expression on cell proliferation, susceptibility to 5-FU, bioenergetic changes, and CEACAM6 expression in CRC cells

**Fig. 2:**
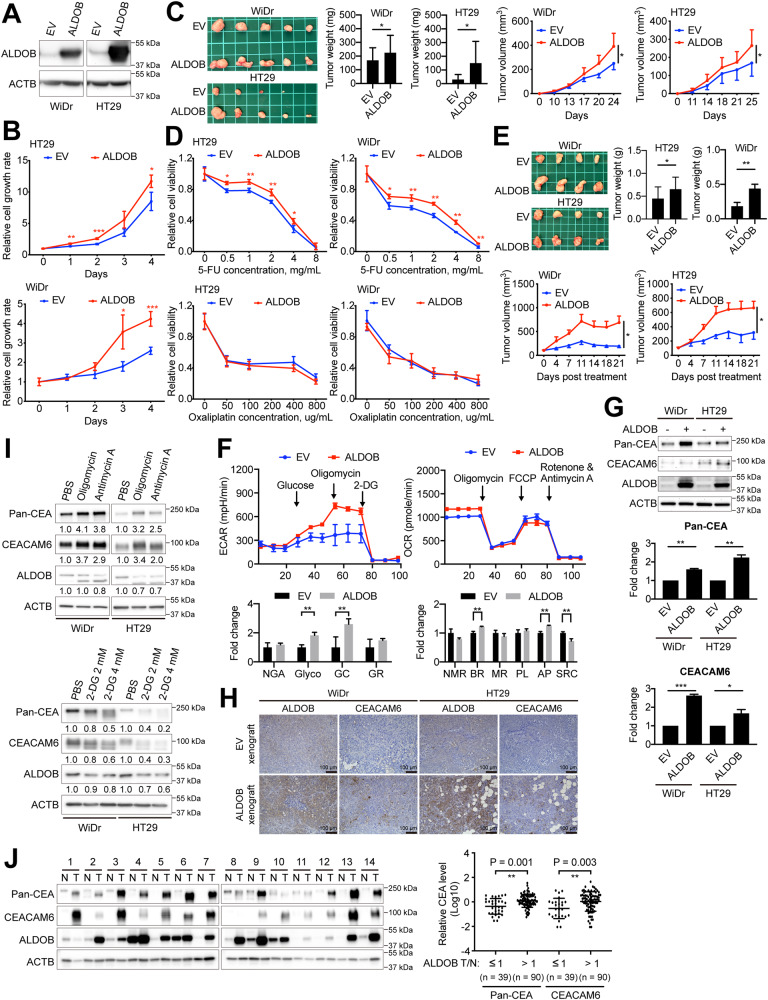
Elevated ALDOB promotes cell growth, 5-FU chemoresistance, CEA expression, and bioenergetic changes in CRC. **A** Representative Western blots showing ALDOB overexpression in CRC, WiDr and HT29 cells. **B** Cell proliferation assay conducted on cells with or without ALDOB overexpression. **C** Left: Xenografts originating from nude mice after subcutaneous injection of cells with or without ALDOB overexpression. Middle: Comparison of tumor weights. Right: Estimated tumor volume over time for each group. Two-tailed unpaired Student’s *t*-test utilized for xenograft comparison. **D** Cell viability response to 5-FU and Oxaliplatin treatment. **E** Upper left: Xenografts derived from nude mice intratumorally injected with 5-FU. Upper right: Comparison of tumor weights. Lower: Estimated tumor volume over time for each group. Two-tailed unpaired Student’s *t*-test used for xenograft comparison. **F** Extracellular acidification rate (ECAR) and oxygen consumption rate (OCR) of cells with or without ALDOB overexpression obtained using the Seahorse assay. **G** Representative Western blots demonstrating CEACEM6 and pan-CEA levels upon ALDOB overexpression in CRC cells. The statistical analysis is shown in the lower panels. **H** Immunohistochemical staining of ALDOB and CEACAM6 in xenograft tissues from (**C**). **I** Representative western blots showing CEACEM6 and pan-CEA levels after the addition of inhibitors of the oxidative phosphorylation (OXPHOS) or the glycolytic pathway in CRC cells. **J** Levels of CEACAM6 and pan-CEA in nontumor and tumor tissues from CRC patients. The statistical analysis is displayed in the right panel. All P values were obtained using the paired two-tailed Student’s *t*-test. *P < 0.05; **P < 0.01; ***P < 0.001.

Previously, it was demonstrated that higher levels of ALDOB expression can boost cell proliferation [19]. Our findings corroborate this idea, as demonstrated in Fig. 2A, B. Moreover, we observed that ALDOB expression enhanced the growth of xenografts, as shown in Fig. 2C. However, it remains unclear whether ALDOB expression has an impact on the sensitivity of CRC cells to commonly used anticancer agents, such as 5-FU and oxaliplatin, in adjuvant chemotherapy. Our results show that while increased ALDOB expression considerably increases the unresponsiveness of CRC cells to 5-FU, it has no effect on the responsiveness to oxaliplatin, as illustrated in Fig. 2D. This reduced drug sensitivity was further validated using a xenograft model, which demonstrated a weakened response to 5-FU in tumors expressing ALDOB, as seen in Fig. 2E.

Given that higher ALDOB expression was associated with increased ECAR and OCR (with borderline significance) in tissues from CRC patients (Fig. 1H, I), we explored its effects on bioenergetic changes. Since ALDOB is an enzyme involved in the glycolytic pathway, we investigated whether its higher expression would enhance aerobic glycolytic activity, specifically the ability to convert glucose to pyruvate (Glyco) and maximal glycolytic capacity (GC), in CRC cells, without any inhibitory effects (Fig. 2F). Surprisingly, we found that increased ALDOB expression led to a modest increase in OCR in CRC cells, especially in basal respiration (BR) and ATP production, despite a decrease in spare respiratory capacity (SRC) (Fig. 2F).

As we observed a correlation between higher tumor ALDOB expression and elevated circulating CEA levels in patients (Table 1), we sought to determine if increased ALDOB expression could promote CEA expression. In CRC, CEA Adhesion Molecule-6 (CEACAM6) is considered the most significant member of the CEA protein family [22]. Therefore, we examined CEACAM6 levels, with a particular focus on CRC cells with and without increased ALDOB overexpression. Our results indicate that higher ALDOB expression significantly increases CEACAM6 and pan-CEA levels in CRC cells, as demonstrated in Fig. 2G. This trend was also evident in xenograft tissues obtained from Fig. 2C, where tumors with ALDOB overexpression exhibited increased CEACAM6 levels (Fig. 2H).

The alteration of CEACAM6 and pan-CEA levels in CRC cells could be a result of ALDOB-induced disruption of bioenergetic homeostasis. To test this hypothesis, we employed inhibitors of either the OXPHOS or glycolytic pathway to induce bioenergetic alterations, and examined their impact on CEACAM6 and pan-CEA levels. Our results, depicted in Fig. 2I upper panel, demonstrate that both CEACAM6 and pan-CEA levels increase when OXPHOS is blocked by either oligomycin or antimycin A. Conversely, the inhibition of the glycolytic pathway by 2-DG led to a concentration-dependent decrease in both CEACAM6 and pan-CEA levels (Fig. 2I lower panel). This suggests that changes in CEACAM6 and pan-CEA levels are downstream consequences of ALDOB-mediated bioenergetic modifications.

To determine if the relationship between ALDOB expression and CEACAM6 or pan-CEA levels is also evident in tissues obtained from CRC patients, we conducted western blotting experiments. Our results, depicted in Fig. 2J, indicate that those with higher ALDOB expression were also associated with increased CEACAM6 and pan-CEA expression in tumors, in agreement with the findings of our cell-based studies.

### ALDOB expression promotes metabolic reprogramming and lactate secretion in CRC to enhance cell proliferation

**Fig. 3:**
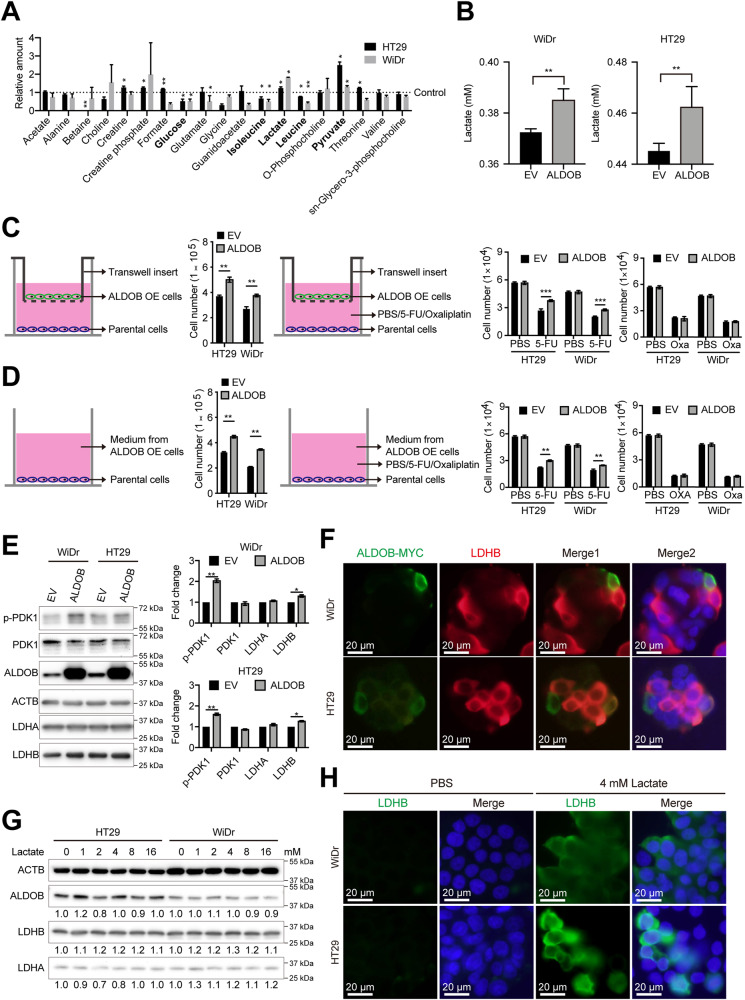
ALDOB-mediated lactagenesis promotes cell growth, 5-FU chemoresistance and LDHB expression through PDK1 activation. **A** Metabolomic analysis based on nuclear magnetic resonance (NMR) showing metabolite levels in CRC cells with or without ALDOB overexpression. Black dashed lines indicate relative metabolite levels in control-treated cells. **B** Lactate levels in medium from cells with control or ALDOB overexpression. **C** Transwell-based co-culture system for assessment of cell growth (left panel) and chemoresistance (right panels). **D** Supplementation of medium from cells with or without ALDOB overexpression for assessment of cell proliferation (left panel) and chemoresistance (right panels). **E** Western blots demonstrating levels of the indicated proteins in cells with or without ALDOB overexpression. The statistical analysis is displayed in the right panel. **F** Immunofluorescence assay showing the expression of exogenous ALDOB (ALDOB-MYC) and LDHB in CRC cells transfected with ALDOB expression plasmid. The scale bar represents 20 μm. **G** Western blots showing the levels of the indicated proteins in cells treated with different concentrations of lactate. **H** Immunofluorescence assay showing LDHB expression in CRC cells treated with indicated concentrations of lactate. The scale bar represents 20 μm. All *P* values were obtained using the paired two-tailed Student’s *t*-test. *P < 0.05; **P < 0.01; ***P < 0.001.

Given ALDOB’s involvement in the glycolytic pathway, we sought to investigate whether its overexpression would impact the metabolic reprogramming of CRC cells. To do this, we used NMR to analyze intracellular metabolite levels. Our results, depicted in Fig. 3A, indicate that ALDOB overexpression led to a significant decrease in glucose, isoleucine, and leucine concentrations, while lactate and pyruvate concentrations were significantly increased. We also examined whether ALDOB overexpression promotes the secretion of lactate, a key metabolite. Figure 3B shows that ALDOB overexpression significantly increased lactate secretion into the culture medium.

To determine whether cell proliferation induced by ALDOB occurs through the secretion of specific factors such as lactate, we conducted two experiments. First, we investigated whether ALDOB-overexpressing cells could secrete agents to promote proliferation and insensitivity to anticancer drugs in parental cells. As shown in Fig. 3C, co-culturing ALDOB-overexpressing cells in transwell chambers significantly promoted parental cell growth and insensitivity to 5-FU, but not oxaliplatin. Second, we assessed whether the culture medium previously used for ALDOB-overexpressing cells contained agents that promote proliferation and insensitivity to anticancer drugs in parental cells. As depicted in Fig. 3D, the medium previously used for ALDOB-overexpressing cells significantly increased parental cell proliferation and insensitivity to 5-FU, but not oxaliplatin.

### ALDOB overexpression induces lactate secretion, leading to increased LDHB expression in adjacent CRC cells

The overexpression of ALDOB has been shown to increase lactate production and secretion (as demonstrated in Fig. 3A, B), but the mechanism by which this occurs remains unclear, as ALDOB is involved in the early steps of the glycolytic pathway, which do not directly lead to higher lactate levels. LDHA has been implicated as a key factor in the conversion of pyruvate to lactate, resulting in the Warburg effect following PDKs activation in cancers [15]. Therefore, levels of activated PDK1 and LDH subunits were examined in cells with and without ALDOB overexpression. Interestingly, no significant changes in LDHA levels were observed, while LDHB and phosphorylated PDK1 levels were found to be increased after ALDOB overexpression (as shown in Fig. 3E). Furthermore, the results of immunofluorescence analysis indicated that the elevated LDHB was primarily derived from neighboring cells adjacent to the cells with increased ALDOB expression (as demonstrated in Fig. 3F). To determine whether this was a result of the ALDOB-mediated increase in secreted lactate, lactate was directly supplemented to the medium, and its effects were examined. As shown in Fig. 3G, LDHB levels increased slightly after exogenous lactate treatment. The immunofluorescence analysis also supported the idea that extracellular lactate promotes LDHB expression in CRC cells (as demonstrated in Fig. 3H).

### Extracellular lactate controls bioenergetic changes and modulates cell proliferation, susceptibility to 5-FU, and CEA expression

**Fig. 4:**
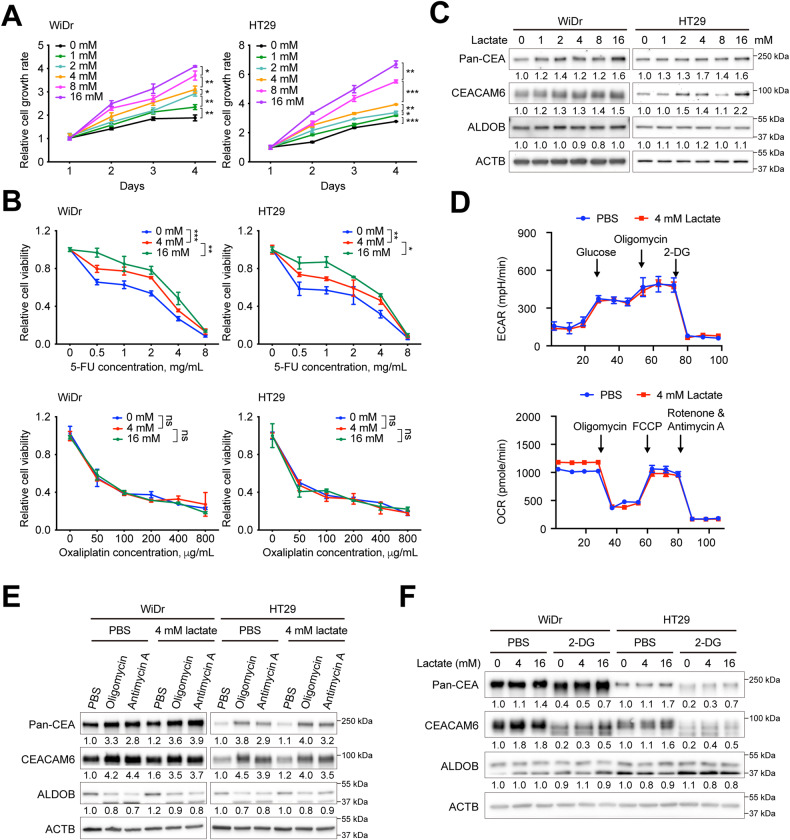
Extracellular lactate promotes cell growth, 5-FU chemoresistance, CEA expression and bioenergetic alterations in CRC. The **A** cell proliferation and **B** cell viability assays were performed using Alarmar blue-based assay. *P* values were obtained using the two-way ANOVA. *P < 0.05; **P < 0.01; ***P < 0.001. **C** Representative Western blots showing the indicated protein levels in cells with different lactate concentrations. **D** Extracellular acidification rate (ECAR) and oxygen consumption rate (OCR) of cells with and without ALDOB overexpression obtained using the Seahorse assay. Representative Western blots showing the indicated protein levels upon simultaneous addition of lactate and inhibitors of **E** the oxidative phosphorylation (OXPHOS) or **F** the glycolytic pathway in CRC cells.

To investigate the role of extracellular lactate in regulating cell proliferation, chemosensitivity, and CEA expression downstream of ALDOB, a series of assays similar to those in Fig. 2 were performed. Figure 4A shows that extracellular lactate promoted cell proliferation in a dose-dependent manner. In addition, at higher lactate concentrations, CRC cells became less sensitive to 5-FU, but not to Oxaliplatin (Fig. 4B). To examine the effect of different lactate concentrations on CEA expression, cells were treated with different lactate concentrations and harvested for analysis. As shown in Fig. 4C, both CEACAM6 and pan-CEA levels increased after extracellular lactate supplementation.

Seahorse assays were conducted to investigate if extracellular lactate directly affects bioenergetic changes. The results are presented in Fig. 4D, indicating that the addition of extracellular lactate enhanced OXPHOS activity, particularly basal respiration, but not aerobic glycolysis. This suggests that the increase in basal respiratory activity observed in Fig. 2D could be attributed to the rise in extracellular lactate induced by the overexpression of ALDOB.

To determine whether extracellular lactate-mediated changes in CEA expression were a result of bioenergetic alterations, experiments were performed to examine the effects of lactate on CEA expression with or without inhibitors for either the OXPHOS or the glycolytic pathway. As shown in Fig. 4E, the addition of OXPHOS inhibitors Oligomycin and Antimycin A significantly increased CEACAM6 and pan-CEA expression. However, no further increase or even a slight decrease in their levels was observed after the simultaneous addition of lactate and the OXPHOS inhibitor, suggesting that lactate-mediated CEA expression was downstream of OXPHOS. On the other hand, co-supplementation with lactate and the glycolytic pathway inhibitor 2-DG partially restored CEACAM6 and pan-CEA levels (Fig. 4F), indicating that lactate-mediated elevation of CEA expression occurred downstream of the glycolytic pathway.

### ALDOB-mediated lactate secretion modulates cell proliferation and susceptibility to 5-FU via CEACAM6 as a downstream effector

**Fig. 5:**
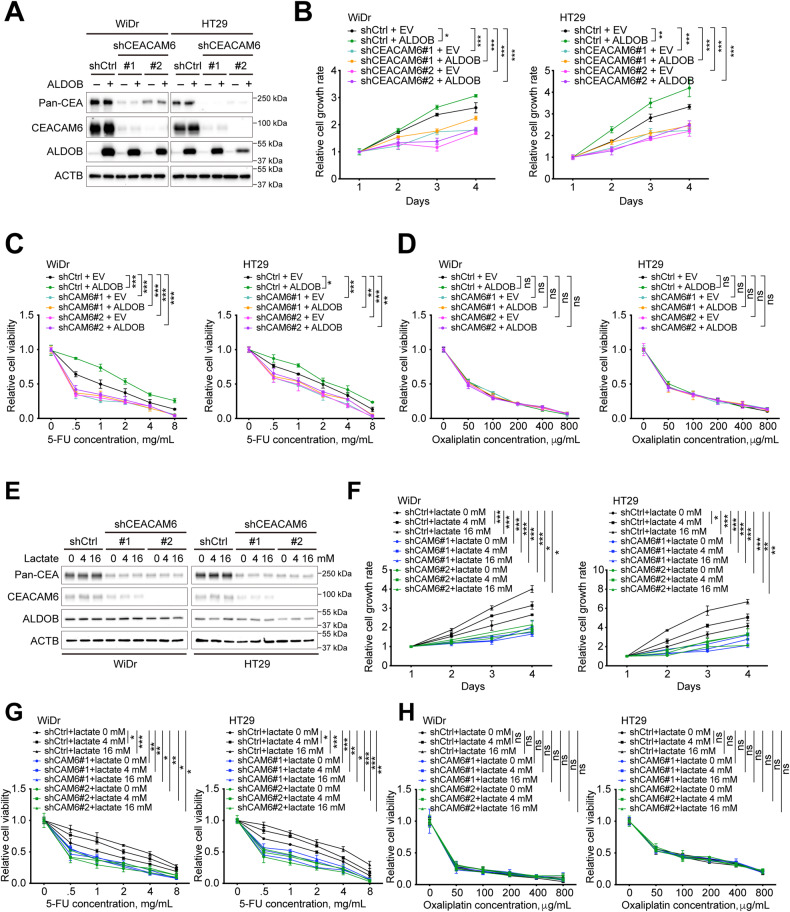
CEACAM6 is an ALDOB/lactate downstream effector regulating cell growth and 5-FU chemoresistance in CRC. **A** Representative Western blots showing the indicated protein levels in CRC cells with or without ALDOB overexpression and CEACAM6 silencing. The **B** cell proliferation and **C, D** cell viability assays were performed using Alarmar blue-based assay. *P* values were obtained using the two-way ANOVA. *P < 0.05; **P < 0.01; ***P < 0.001. **E** Representative Western blots showing the indicated protein levels in CRC cells with or without lactate treatment and CEACAM6 silencing. The **F** cell proliferation and **G**, **H** cell viability assays were performed using Alarmar blue-based assay. All *P* values were obtained using the two-way ANOVA. *P < 0.05; **P < 0.01; ***P < 0.001.

To investigate if CEACAM6 is a potential downstream factor of ALDOB in regulating cell behavior, proliferation, and chemosensitivity, assays were performed with and without concurrent ALDOB overexpression and silencing of CEACAM6 (Fig. 5A). Notably, silencing CEACAM6 had an impact on pan-CEA levels. It was observed that silencing CEACAM6 significantly reduced the proliferation and sensitivity of CRC cells to 5-FU, but not to oxaliplatin, even under ALDOB overexpression (Fig. 5B–D). These findings suggest that CEACAM6 is a downstream effector of ALDOB.

In order to investigate whether CEACAM6 also functions downstream of extracellular lactate in regulating CRC cell proliferation and susceptibility to 5-FU, assays were conducted with and without coexistence of extracellular lactate and CEACAM6 silencing (Fig. 5E). The results showed that silencing of CEACAM6 significantly decreased cell proliferation capacity and sensitivity to 5-FU, regardless of the presence of extracellular lactate (Fig. 5F–H). These findings suggested that CEACAM6 acted as a downstream factor of extracellular lactate in modulating CRC cell behavior.

### ALDOB-induced lactate increase facilitates CEACAM6 stability via augmented lysine lactylation

**Fig. 6:**
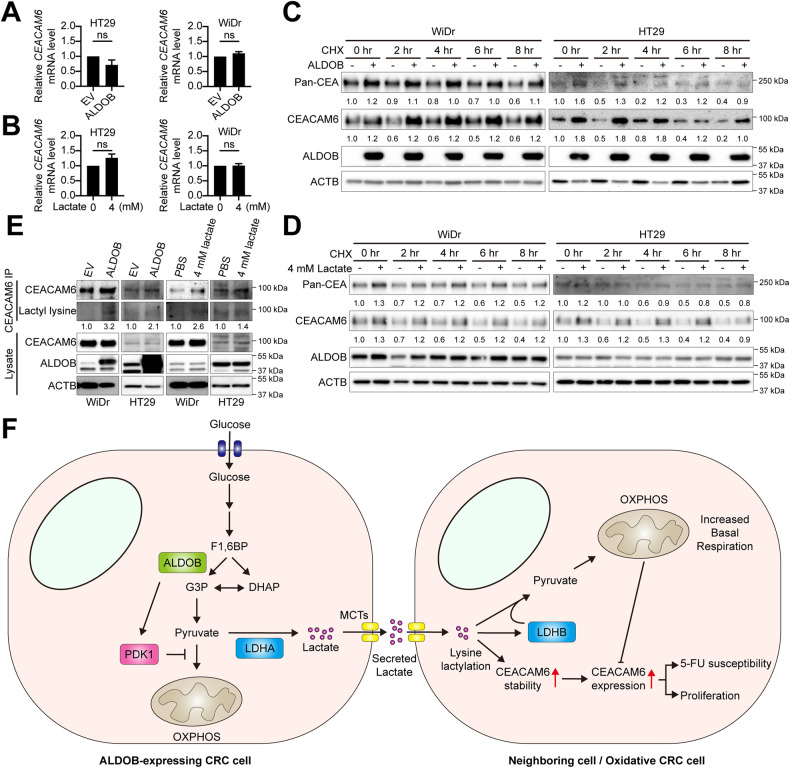
ALDOB-mediated lactagenesis boosts CEACAM6 expression by enhancing protein stability through inducing lysine lactylation. **A,** **B** Statistical analysis of relative CEACAM6 mRNA levels in cells with and without ALDOB overexpression or lactate treatment. *P* values calculated using the two-tailed paired Student’s *t*-test. *,*P* < 0.05; **P < 0.01; ***P < 0.001. **C**, **D** Representative Western blotting illustrating indicated protein levels in CRC cells with or without ALDOB overexpression or lactate treatment under cycloheximide (CHX) treatment. **E** Representative Western blotting showing indicated protein levels in CEACAM6 immunoprecipitates or lysates. **F** The working model proposed in this study. High ALDOB expression in CRC cells activates PDK1, which inhibits the entry of pyruvate into oxidative phosphorylation (OXPHOS) and enhances lactate conversion by LDHA. The excess lactate is then secreted into the tumor microenvironment and taken up by neighboring cells, inducing the expression of LDHB and CEACAM6. For CEACAM6, it is possible that the elevation is through lysine lactylation. LDHB increases the conversion of lactate to pyruvate, enabling neighboring cells to become OXPHOS-dependent. CEACAM6, as an ALDOB/lactate downstream effector, regulates cell growth and 5-FU chemoresistance in CRC.

To delve into the potential mechanism behind ALDOB-mediated lactagenesis in activating CEACAM6 expression, we tested whether ALDOB overexpression and exogenous lactate treatment influenced CEACAM6 mRNA levels, subsequently leading to increased CEACAM protein levels. However, our findings indicated that neither ALDOB overexpression (Fig. 6A) nor lactate treatment (Fig. 6B) consistently stimulated *CEACAM6* mRNA expression, suggesting that the elevation in CEACAM6 protein level did not arise from augmented mRNA levels.

Recent studies have highlighted a novel protein post-translational modification known as lysine lactylation (Kla), which has been recognized as a hallmark of metabolic reprogramming. This modification plays a pivotal role in balancing gene expression and metabolic reprogramming in cancers, potentially through influencing protein stability [23, 24]. In light of this, we put forth the hypothesis that ALDOB-mediated lactogenesis might activate CEACAM6 through Kla. To scrutinize this hypothesis, we collected cells with and without ALDOB overexpression and treated them with cycloheximide, an agent that impedes protein translation, followed by performing western blotting. As depicted in Fig. 6C, our results revealed that after the inhibition of new protein synthesis by cycloheximide, CEACAM6 protein levels gradually declined over time in the control group. In contrast, in cells overexpressing ALDOB, this reduction was notably attenuated. A similar trend was evident in cells treated with exogenous lactate, as illustrated in Fig. 6D. These observations suggest an enhanced protein stability of CEACAM6 following ALDOB overexpression and lactate treatment.

Intriguingly, to ascertain whether the increased protein stability could be linked to the state of Kla within CEACAM6, we conducted an immunoprecipitation of CEACAM6 followed by the detection of Kla using a specific antibody. Our findings, illustrated in Fig. 6E, demonstrated that elevated Kla levels were observed in precipitates from lysates of cells overexpressing ALDOB and treated with lactate. These compelling results imply that ALDOB-mediated lactagenesis promotes the activation of CEACAM6 through the augmentation of Kla, subsequently enhancing CEACAM6 protein stability.

## Discussion

The ALDOB protein level has been suggested as a biomarker for predicting postoperative prognosis in CRC patients, as well as the risk of colon cancer liver metastases and the response of rectal cancer patients to neoadjuvant chemoradiation therapy [19–21]. Additionally, previous studies have demonstrated that increased ALDOB expression can stimulate CRC cell proliferation and migration [19]. While the regulation of EMT has been proposed as a mechanism for controlling cell migration in cells with reduced ALDOB expression, our results (Figure S2) did not support this theory, likely due to the use of different cell lines. However, it remains unclear how ALDOB regulates CRC cell proliferation activity. We discovered that patients with high tumor expression of ALDOB were associated with high levels of circulating CEA (Table 1), prompting us to investigate whether CEACAM6 expression was correlated with ALDOB levels. Our findings revealed that this correlation was mediated by the disruption of ALDOB levels and the glycolytic pathway (Fig. 2F–I).

The oncogenic properties of glycolytic pathway enzymes, such as their association with cell growth, migration, and chemosensitivity, have been studied extensively in various types of cancer [25]. Similarly, CEA expression has been identified as a biomarker for predicting cancer incidence, especially for gastric cancer and CRC, with the ability to promote tumor migration, invasion, metastasis, and angiogenesis [26–28]. However, the relationship between the expression of enzymes in the glycolytic pathway and CEA levels in CRC remains unclear. Moreover, it is not well understood how CEA expression is regulated. In our study, we utilized antibodies against both CEACAM6 (the predominant form of CEA in the protein family in CRC [22]) and pan-CEA to demonstrate that they are downstream effectors of ALDOB and/or aerobic glycolysis, which regulate cell proliferation and chemosensitivity by affecting glycolysis-mediated lactate production and secretion. Our findings suggest that increased aerobic glycolytic capacity may be the primary cause of elevated CEACAM6 levels in the blood or CRC, providing new insights into the regulation of CEA levels in CRC patients.

Lactagenesis in lactagenic cancer cells is believed to be the primary cause and purpose of the Warburg effect [29]. There is emerging evidence to suggest that secreted lactate, an oncometabolite, can modulate cancer incidence, progression, and susceptibility to cancer treatment by promoting TME acidification [17]. To investigate the argument in favor of extracellular lactate-mediated effects on cell growth and chemosensitivity, we supplemented an acidified medium with lactate and found that it did not improve the acidification of the medium. Interestingly, the pH value increased after high lactate supplementation (Figure S3), indicating that the lactate-mediated promotion of cell proliferation and chemosensitivity was independent of TME acidification. Moreover, our findings suggest that the entry of extracellular lactate into cells promotes intracellular LDHB expression and further affects CRC bioenergetic changes. In summary, it is hypothesized that once secreted lactate enters cells, it can promote cell growth and chemosensitivity by modulating bioenergetic alterations through LDHB-mediated changes.

To summarize, this study has demonstrated that overexpression of ALDOB in CRC cells promotes lactagenesis by regulating PDK1 activation. The secreted lactate is then transported to neighboring cells and converted to pyruvate by lactate-induced LDHB, enhancing the ability of OXPHOS in terms of basal respiration and acting as a repressor of CEACAM6 expression. Consequently, ALDOB/lactate-mediated expression of CEACAM6 promotes cell proliferation and 5-FU chemoresistance in CRC cells (Fig. 6F). These findings shed light on the Warburg effect in the CRC TME, particularly with respect to the involvement of CEA downstream of the Warburg effect.

## Materials and methods

### Patients and samples

This study retrospectively enrolled two cohorts: cohort-1, which consisted of 428 CRC patients who underwent surgical resection, and cohort-2, which included 64 patients with AP. Of the patients in cohort-1, 299 were available for paraffin-embedded tissue sections, while the remaining 129 were available for frozen tissue. In cohort-2, all 64 patients were available for frozen tissue analysis. The IHC staining was performed on paraffin-embedded tissues, while RNA isolation, Western blot, and seahorse analysis were conducted on frozen tissues. All patient-derived tissues were retrieved from the Tissue Bank of Linkou Chang Gung Memorial Hospital, and their clinical data were reviewed, including gender, age, height, weight, body mass index (BMI), tumor location, tumor differentiation status, local invasion score, maximum tumor size, circulating CEA level, history of adjuvant chemotherapy, tumor-to-serosa distance, date of CRC diagnosed, date of CRC-related death, date of last follow-up, and date of recurrence or metastasis. This study was conducted with the approval of the Chang Memorial Hospital Institutional Review Board (Approval number: 202001598B0 and 202102161B0).

### Western blot analysis and immunoprecipitation

Western blots were conducted following previously described methods [30]. The rabbit polyclonal antibodies against ALDOB (Proteintech, Rosemont, IL, USA, Cat. 18065-1-AP, diluted 1:5000), LDHA (Proteintech, Cat. 21799-1-AP, diluted 1:1000), and LDHB (Proteintech, Cat. 14824-1-AP, diluted 1:5000), along with the rabbit monoclonal antibody against ACTB (Sigma-Aldrich, St. Louis, MO, USA, Cat. A5441, diluted 1:10000), PDK1 (Cell Signaling Technology, Danvers, MA, USA, Cat. #5662, diluted 1:1000), phospho-PDK1 (Cell Signaling Technology, Cat. #3438, diluted 1:1000), and lactyl-lysine (Cat. PTM-1401RM, PTM BIO, Chicago, IL, USA), and the mouse monoclonal antibodies against pan-CEA (Santa Cruz Biotechnology, Dallas, TX, USA, Cat. Sc-48364, diluted 1:100), and CEACAM6 (Santa Cruz Biotechnology, Cat. sc59899, diluted 1:100) were used for Western blot analysis. Lysate preparation and immunoprecipitation were performed according to previously established methods [31]. In each reaction, 1 μg of the mouse monoclonal antibody against CEACAM6 (Santa Cruz Biotechnology, Cat. sc59899) was employed. After thorough washing, the immunoprecipitates were utilized for subsequent western blot analysis.

### IHC staining

IHC was conducted following previously described methods [32]. A rabbit monoclonal antibody against ALDOB (Proteintech, Rosemont, IL, USA, Cat. 14824-1-AP) and CEACAM6 (Santa Cruz Biotechnology, Cat. sc59899) were used for staining at dilutions of 1:200 and 1:50, respectively. The intensity of the staining signal was quantified using Image J (National Institutes of Health, Bethesda, MD, USA, Fiji edition) and subsequently analyzed.

### RNA extraction, cDNA synthesis and real-time quantitative polymerase chain reaction (RT-qPCR)

Total RNA isolation was conducted following previously described methods [33]. The PrimeScript RT Reagent Kit (TaKaRa, Shiga City, Japan, Cat. RR037A) was utilized for first-strand cDNA synthesis, using up to 0.5 μg of total RNA in accordance with the manufacturer’s instructions. The real-time PCR system QuantStudio 5 was used for subsequent analysis. In this study, the following primers were used for RT-qPCR: ACTB_qPCR_F: CACCAACTGGGACGACATGG, ACTB_qPCR_R: AGGATCTTCAGAGGTAGTC, ALDOB_qPCR_F: TCTTCTCTGTGGACAGTTCCA, ALDOB_qPCR_R: TCCTTGGTCTAACTTGATTCCCA, CEACAM6_qPCR_F: TCAGCCACTGGCCTCAATAG, and CEACAM6_qPCR_R: TCTGGTCCAATCTGCCAGTC.

### Seahorse assay

Simultaneous measurements of OCR and ECAR of patient tissues or cultured cells were performed using a Seahorse XF24 analyzer (Agilent, Santa Clara, CA, USA) as formerly described [34]. In brief, freshly isolated tissue was rinsed with assay medium (unbuffered Dulbecco’s modified Eagle medium (DMEM) (pH 7.4)) and sliced into 1 mm pieces. Equal amounts of tissue specimens were placed in each well of an XF24 Islet Capture Microplate (Agilent, Cat. 101174-100) and covered with an islet capture screen to allow free perfusion while minimizing tissue movement. We added 500 μl of assay medium to each well, incubated the microplate at 37 °C in a non-CO_2_ incubator for 30 min, and then used the Seahorse XF24 analyzer to measure OCR and ECAR simultaneously. During the measurement, the tissue was not pushed by the sensor, and we used at least three replicates from each tissue for the assays. We transferred the specimen from the colonoscopy suite or operating theater to the Seahorse platform within two hours. To detect bioenergetic alterations in cells, we seeded cells into 24 wells of an XF24-cell culture microplate at a density of 7 × 10^4^ or 5 × 10^4^ cells/well and incubated them in a 5% CO2/air atmosphere at 37 °C for 24 h. We then measured OCR and ECAR under basal conditions using the Mito Stress test Kit (Agilent, Cat. 103015-100) to reveal key parameters of the metabolic function of cells. OCR was measured under basal conditions and in the presence of the ATP synthase inhibitor oligomycin (1 μM), the mitochondrial uncoupler carbonyl cyanide-4-(trifluoromethoxy)-phenyl-hydrazone (FCCP; mitochondrial respiration uncoupler, 0.5 μM), and the respiratory chain inhibitor antimycin A (0.5 μM). We used the Glycolysis Stress test Kit (Agilent, Cat. 103020-100) to measure ECAR under various conditions. We made three baseline recordings, followed by sequential injection of glucose (10 mM), the ATP synthase inhibitor oligomycin (1 μM), and the glycolysis inhibitor 2-deoxy-d-glucose (2-DG; 100 mM). We performed the assays in at least three replicates and presented OCR and ECAR as absolute values, normalized by the tissue protein concentration (mMol/min/mg for OCR, whereas mpH/min/mg for ECAR) or cultured cell concentration (pMol/min/μg for OCR, whereas mpH/min/μg for ECAR).

### Cell culture and transfection

HT29 (CVCL-0320) and WiDr (CVCL-2760) cell lines were used in this study and maintained in RPMI-1640 and DMEM media, respectively, at standard culture conditions of 5% CO2 in a humidified 37°C incubator. Both cell lines were routinely screened to ensure they were free of mycoplasma contamination. To express ALDOB with an N-terminal myc tag, a plasmid was purchased from SinoBiological (Beijing, China, Cat. HG13207-NM). For transient expression of ALDOB, cells were seeded 16 h prior to transfection, and 5 μg of plasmid DNA was used for transfection in 6 cm plates. MaestroFectin (Omics Bio, New Taipei City, Taiwan, Cat. MF002) transfection reagent was used according to the manufacturer’s instructions.

### Oligomycin, antimycin, 2-deoxyglucose (2-DG), and lactate treatment

To block OXPHOS activity, oligomycin (Cell Signaling Technology, Danvers, MA, USA, Cat. #9996) and Antimycin A (Sigma-Aldrich, St. Louis, MO, USA, Cat. A8674) were used at a final concentration of 1 μM and 2 μg/mL, respectively. The glycolytic pathway was blocked using 2-DG (Sigma-Aldrich, Cat. D8375) at a final concentration of 2 or 4 mM. To mimic extracellular secreted lactate, lactate (Sigma-Aldrich, Cat. L7022) was used. All cells were collected for subsequent analysis 48 h after treatment.

### Cell growth and viability assay

Cell proliferation rate was evaluated according to previously described methods [35]. Specifically, 3 × 10^3^ cells were seeded into each well of a 96-well plate and incubated for 24 h before adding Alamar Blue cell viability reagent (Invitrogen, Waltham, MA, USA, Cat. DAL1100) directly to the medium. Fluorescent metabolite quantification assays were performed daily for up to 4 days. To assess cell viability in response to anticancer drugs, 1 × 10^4^ cells were seeded per well of a 96-well plate at least 16 h prior to treatment with 5-FU or Oxaliplatin. Following a 24 h incubation at 37 °C, the medium in each well was refreshed, and Alamar Blue cell viability reagent was added. Fluorescent metabolite quantification was performed after a further 3 h of incubation at 37 °C. The growth-promoting and anticancer drug insensitivity effects of ALDOB were also assessed using a transwell-based co-culture system and culture of parental cells in medium containing ALDOB-overexpressing cells. For the transwell-based co-culture system, 2 × 10^4^ parental cells were seeded at the bottom of each well of a 24-well plate, and 1 × 10^4^ cells were seeded in the transwell chamber with or without ALDOB overexpression. After 48 h of co-cultivation, the cells in the bottom were suspended with trypsin, and the total number of cells was counted. Similarly, 2 × 10^4^ parental cells were seeded in each well of a 24-well plate and the next day, replaced with fresh medium containing medium derived from control or ALDOB-overexpressing cells previously used. After 48 h of co-cultivation, the cells were suspended with trypsin, and the total number of cells was counted.

### Xenograft tumor growth and 5-FU treatment

In this study, 36 male nude mice (6 weeks old) purchased from National Animal Center (Taiwan) were used. Of these, 20 were dedicated to the xenograft tumor growth study, with 10 for the WiDr cell group and 10 for the HT29 cell group. Subcutaneous injection of approximately 5 × 10^6^ cells, either with or without ALDOB overexpression, was conducted. Tumor length and wide were measured twice weekly until sacrifice. The formulation: ${\rm{V}}=0.5\times {\rm{Length}}\times {{\rm{Wide}}}^{2}$ was employed for tumor volume calculation. Upon sacrifice, tumors were excised, weighed, and evaluated. For 5-FU treatment, intratumoral injection of 5 mg/kg 5-FU was initiated when xenografts reached an estimated volume of 70–120 mm^3^, administered twice weekly. After six 5-FU injections, mice were sacrificed, tumors were removed, weighed, and fixed in 10% formaldehyde. Paraffin embedding followed for subsequent IHC analysis. Animal protocols adhered to regulations set by the Animal Ethics Committee of Linkou Chang Gung Memorial Hospital.

### Metabolite analysis

The intracellular metabolites were analyzed using nuclear magnetic resonance (NMR), following a previously described protocol [36]. To prepare the samples, approximately 3 × 10^6^ cells from a 10 cm culture dish were harvested and subjected to metabolite extraction in the aqueous phase. The cells were lysed with pre-chilled methanol and then mixed with chloroform and sterile water at a 1:1:1 ratio. After centrifugation, the aqueous phase was collected and concentrated using a vacuum concentrator with a lyophilizer. The lyophilized metabolites were then redissolved in NMR buffer containing a chemical shift reference. The samples were placed in a 4-inch SampleJet NMR tube and analyzed using a Bruker Avance III HD spectrometer with a 14.1-T magnet for 1H 600 MHz. Independent biological triplicates were performed for reproducibility. The acquisition and processing of data were performed using Topspin 3.6, and the experiments were automated using the IconNMR program with a standard pulse sequence (cpmgpr1d). Continuous wave irradiation was used with 25 Hz RF during the relaxation delay and mixing time, with a magnetic field Z-gradient applied for 1 min. The relaxation delay was set to 4 s, the mixing time was 10 min, and the acquisition time was 2.7 s with 128 transients of 64,000 data points recorded. The receiver gain was set to 64, and a cooling rack was used to maintain the sample at 283 K.

### Immunofluorescence assay (IFA)

The protocol for IFA was performed with minor adjustments as previously described [37]. In brief, cells were seeded on coverslips and either transfected with an ALDOB expression plasmid or treated with lactate. After 48 h of incubation, cells were fixed using 4% paraformaldehyde (Sigma, Cat. 16005). A mouse monoclonal antibody targeting the MYC tag (Proteintech, Cat. 60003-2-Ig), and a rabbit monoclonal antibody to LDHB (Proteintech, Cat. 14824-1-AP) were used at a 1:200 dilution. Goat antibodies to rabbit or mouse IgG labeled with fluorescein (FITC) or rhodamine (TRITC) were used as secondary antibodies at a 1:200 dilution.

### Lentiviral-mediated silencing of CEACAM6

Lentiviral-mediated transduction of shRNA against CEACAM6 was carried out using a previously described protocol with slight modifications [38]. In brief, 293 T cells (CVCL_0063) were used to package virions by transfecting 1.3 μg of shRNA, 1.1 μg of pCMV-ΔR8.91, and 0.1 μg of pMD.G. After 24 h of incubation, the medium was replaced with 1% bovine serum albumin (BSA)-containing medium and further incubated for 48 h. The medium was then collected and centrifuged, and the lentivirus-containing supernatant was passed through a 0.22 μm pore size filter and aliquoted for future experiments. For transduction, cells were seeded and incubated for 16 h before adding the virions directly into the culture dish containing a fresh medium with 8 μg/mL polybrene (Sigma, St. Louis, MO, USA, H9268). After 48 h of incubation, cells were selected using 2 μg/mL puromycin (Gibco, Waltham, MA, USA, Cat. A1113803) and allowed to stabilize for two generations before subsequent analysis. The shRNA sequences used to silence CEACAM6 were 5′-CCAACATCACTGTGAATAATA-3′ (clone ID: TRCN0000062299) for #1 and 5′-ATCAGATCTATCTTGTCAATC-3′ (clone ID: TRCN0000412385) for #2. The control used in this assay was shRNA against LacZ, 5′-GCCGTCGTATTACAACGTCGT-3′ (clone ID: TRCN0000231700). All shRNA clones were obtained from the RNAi core, Academia Sinica, Taiwan.

### Statistical analysis

The parametric data were reported as mean ± standard deviation and compared using a two-sample *t*-test. Dichotomized data were presented as numbers and percentages (%) and compared using the chi-square test. Univariate and subsequent multivariate Cox proportional hazard models were used to assess the prognosis for clinical factors and ALDOB expression. Significant factors identified in the univariate analysis were included in the subsequent multivariate Cox proportional hazards analysis. Survival probabilities between different groups were estimated using the Kaplan–Meier method, and the log-rank test was applied. All tests were two-tailed, and statistical significance was set at *P* < 0.05. The Statistical Package for the Social Sciences (SPSS) statistics Version 20 was used to perform all statistical analyses.

### Supplementary information

Checklist

Original Data File

Supplemental material

## Data Availability

The authors declare that all relevant data of this study are available within the article and its supplementary files or from the corresponding author on reasonable request.
