# Supplementary material for: Aldolase B-driven lactagenesis and CEACAM6 activation promote cell renewal and chemoresistance in colorectal cancer through the Warburg effect
Source: Cell Death Dis. 2023 Oct 10;14(10):660. doi: 10.1038/s41419-023-06187-z (PMC10564793; doi:10.1038/s41419-023-06187-z)

Raw image for Figure 1C

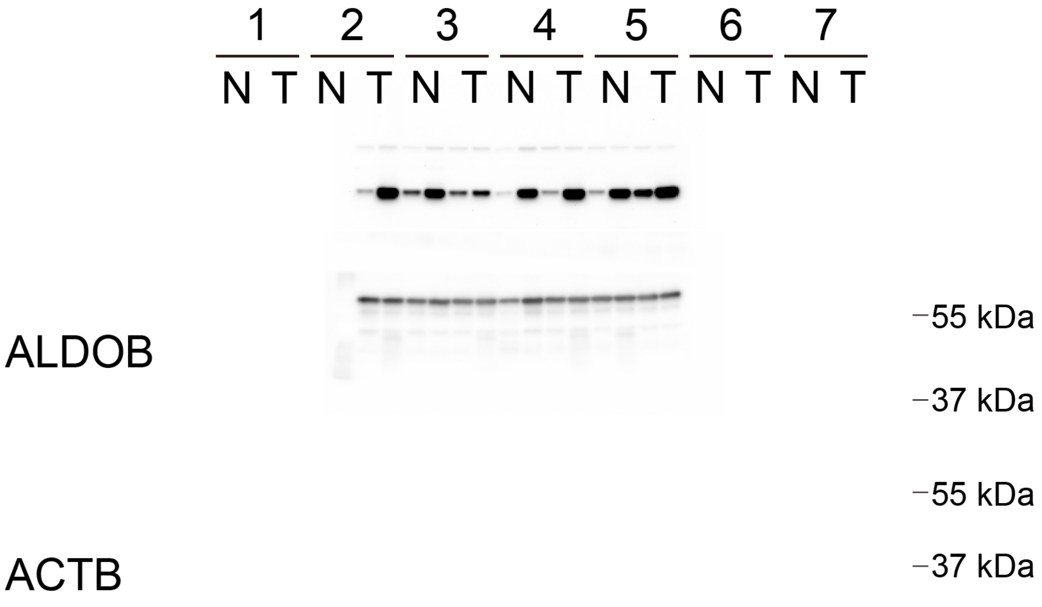

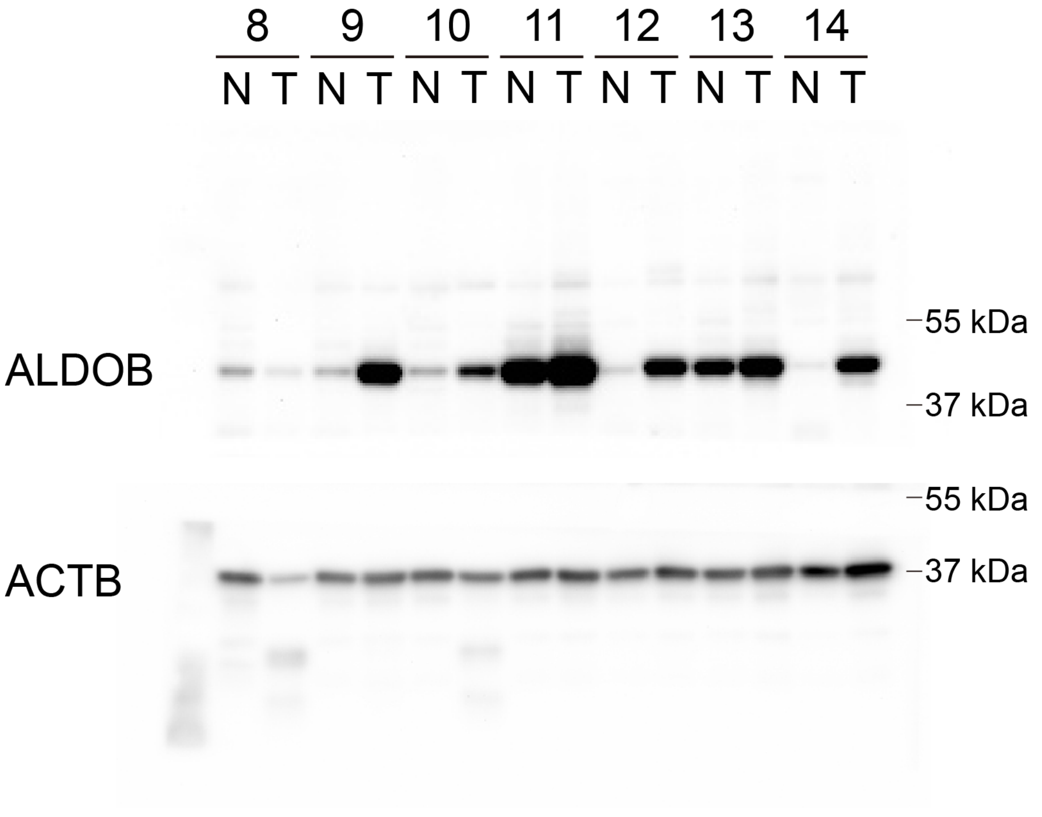

Raw image for Figure 2A

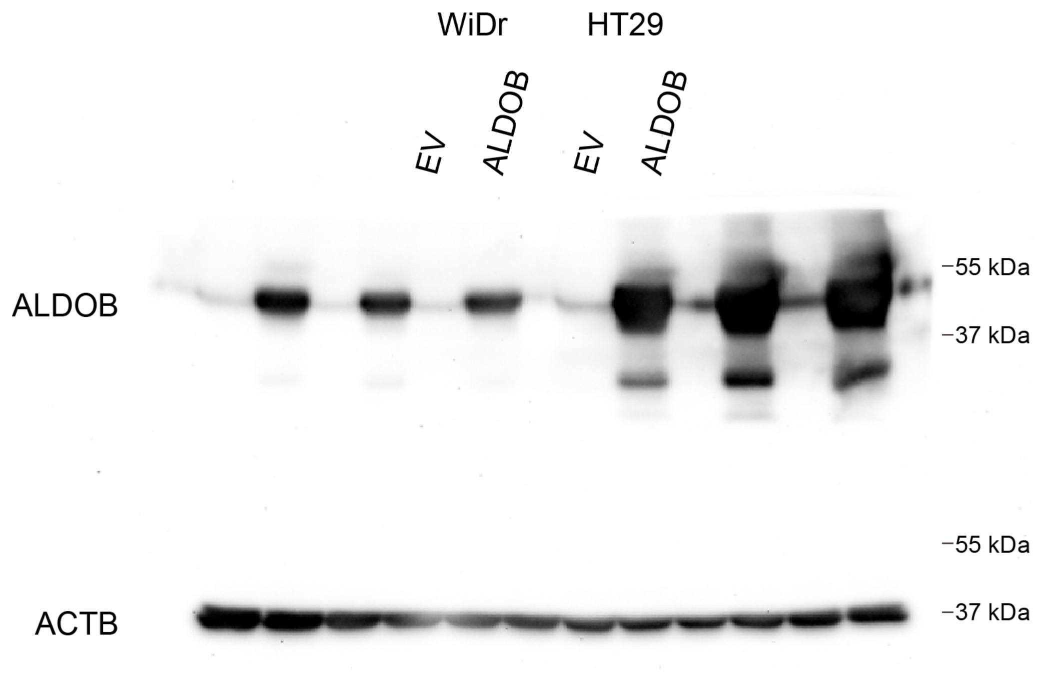

Raw image for Figure 2E

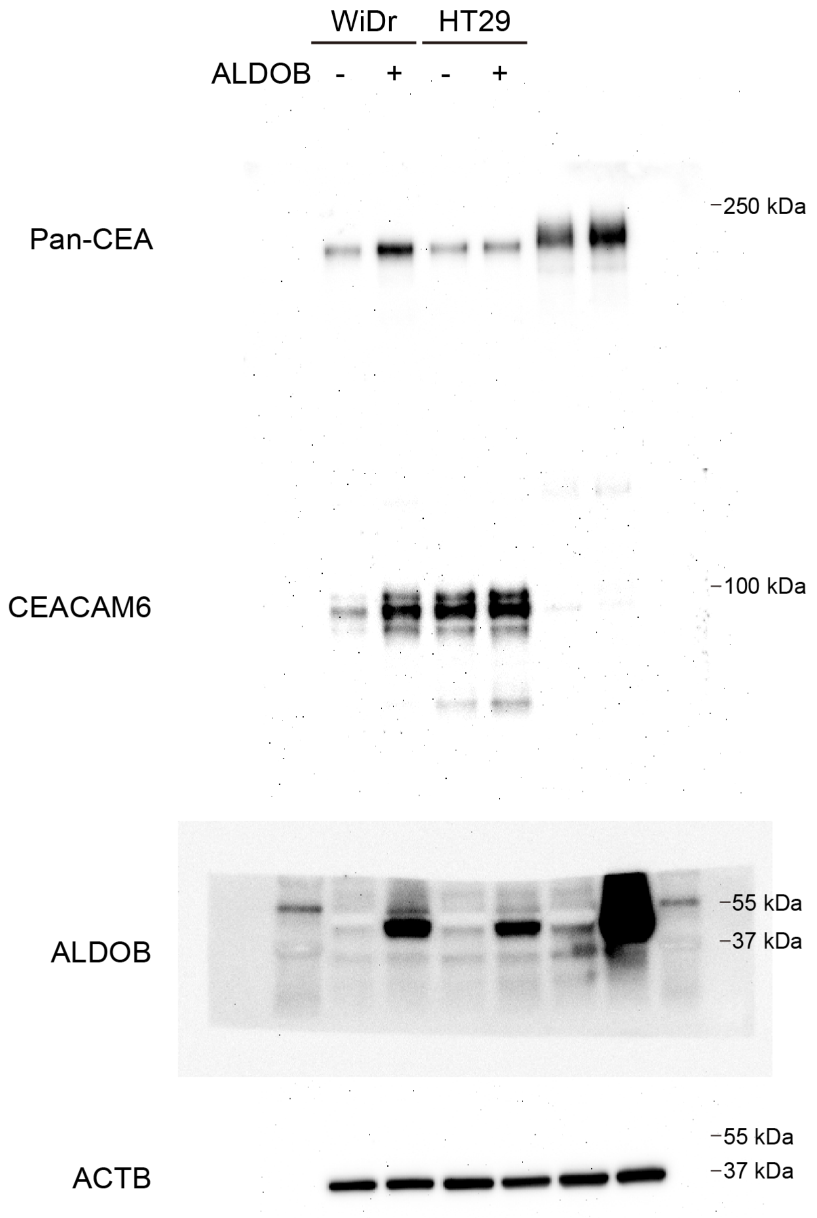

Raw image for Figure 2F

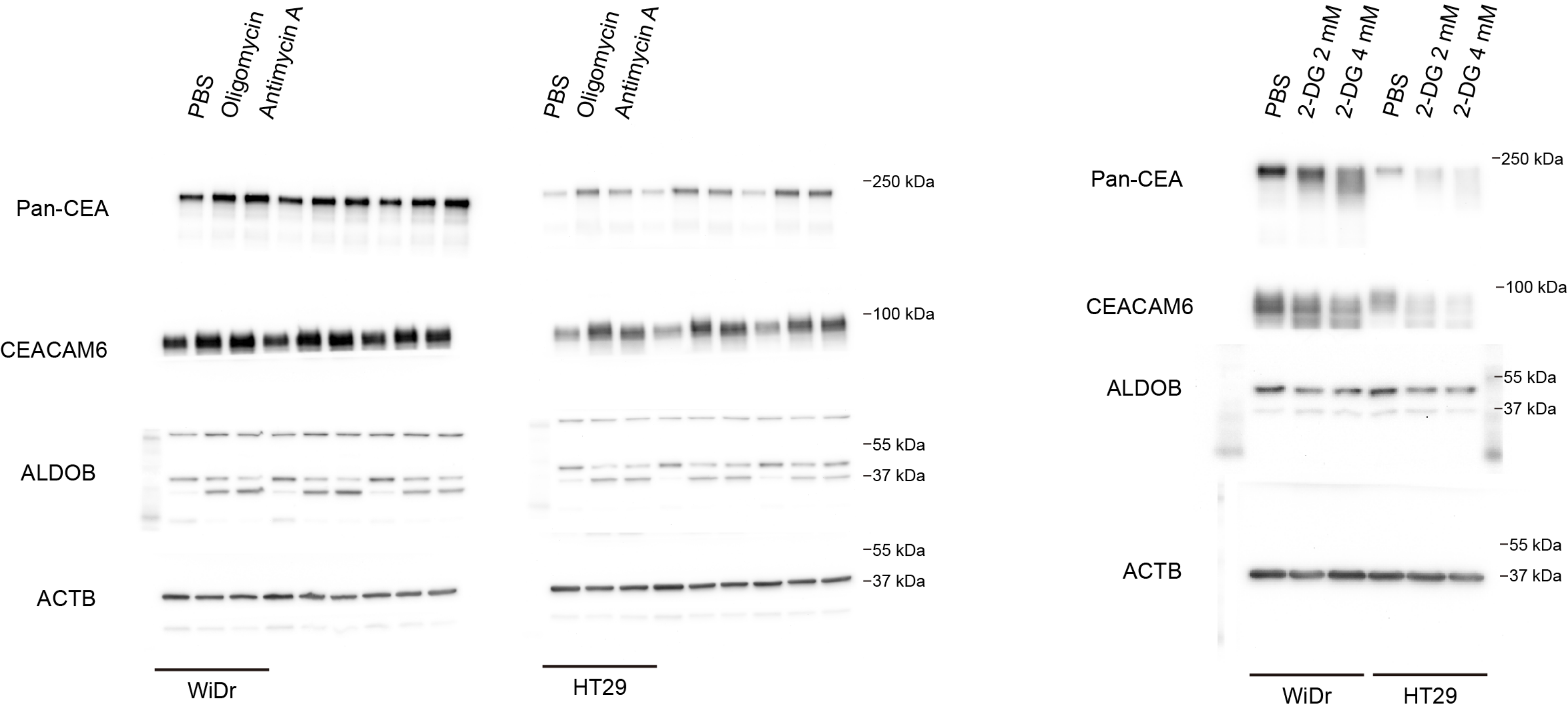

Raw image for Figure 2G

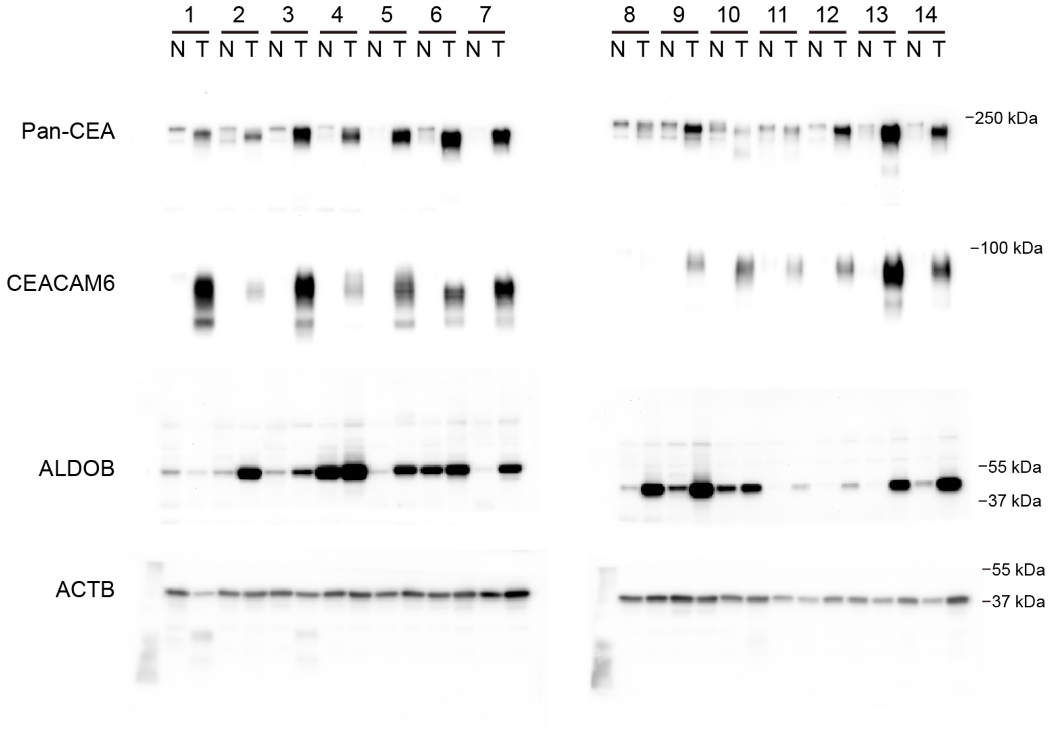

Raw image for Figure 3E

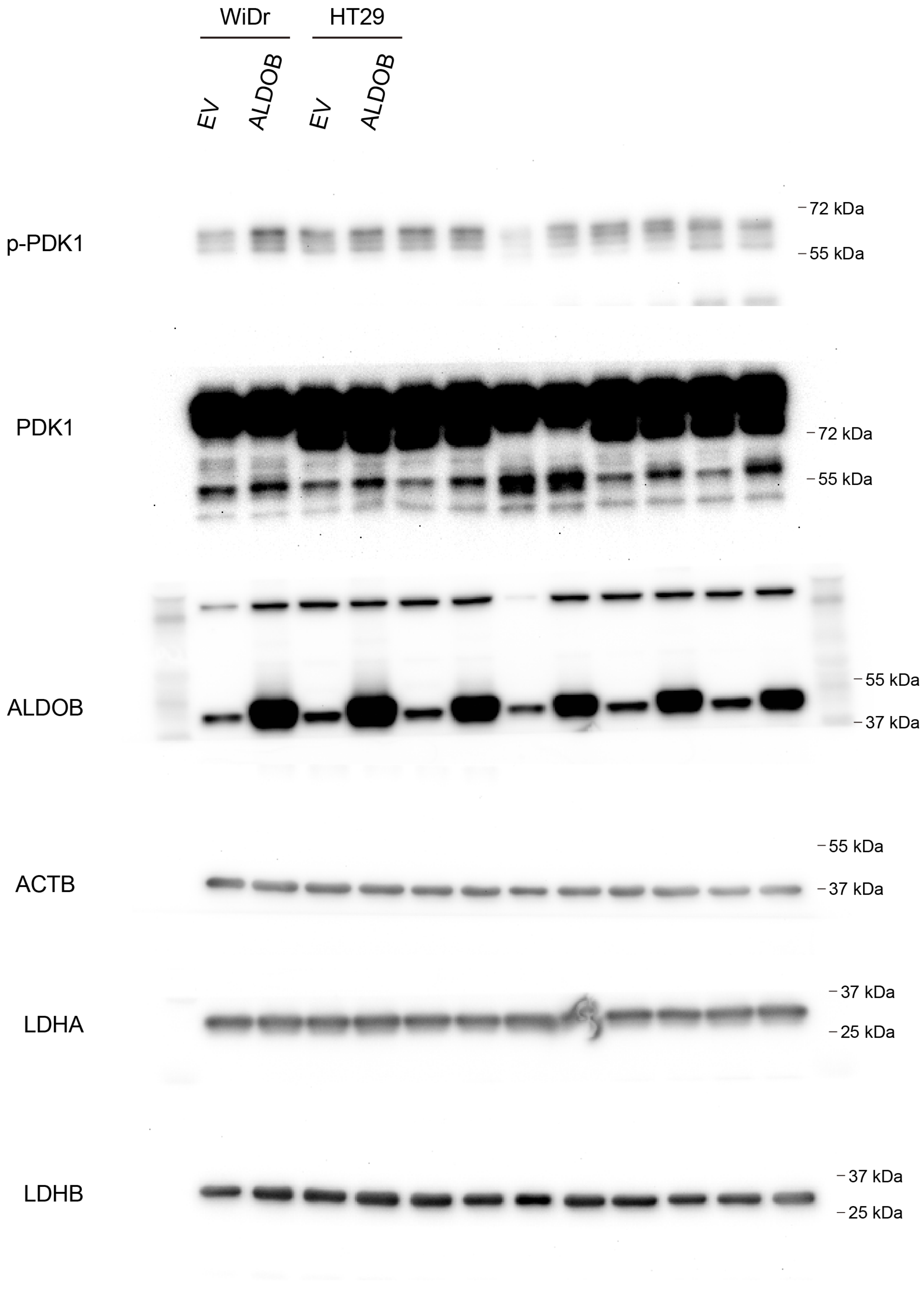

Raw image for Figure 3F

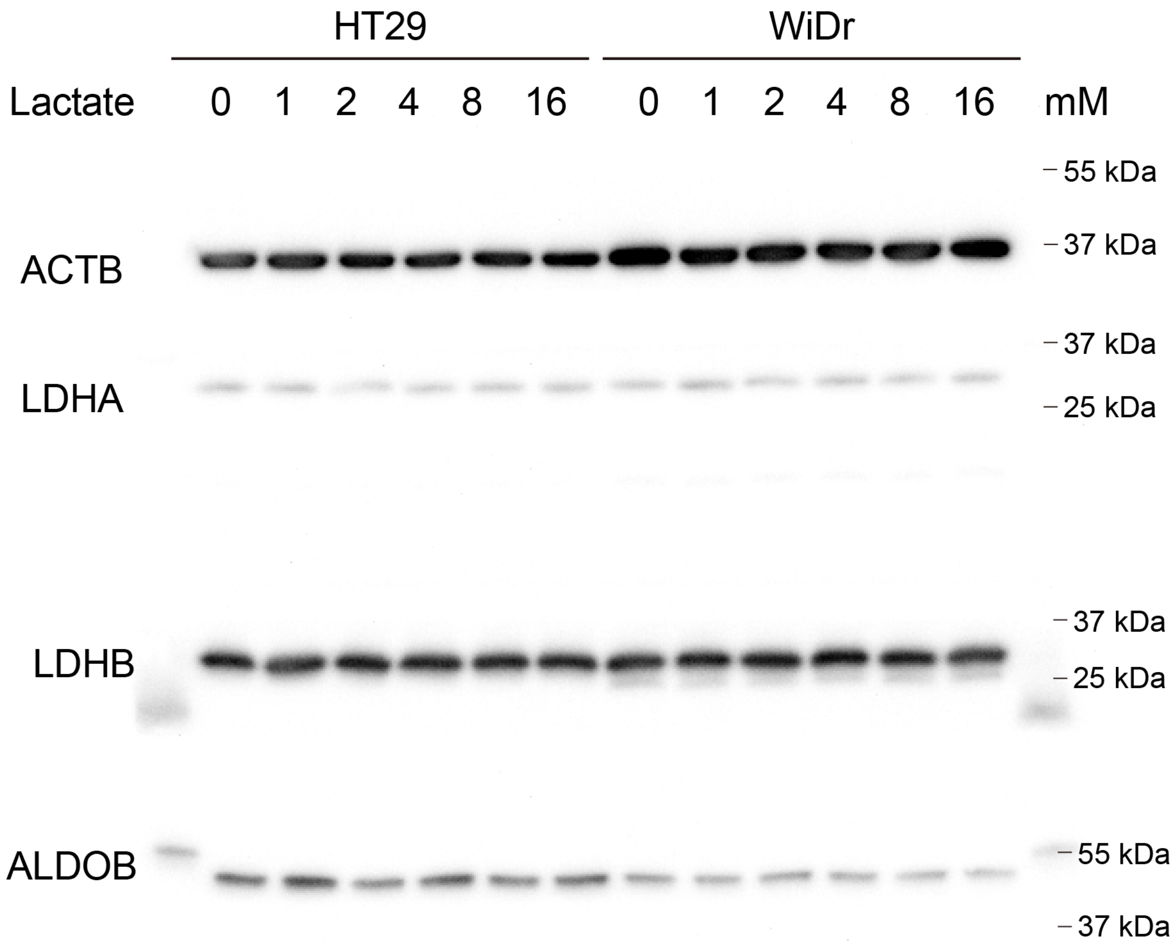

Raw image for Figure 4C

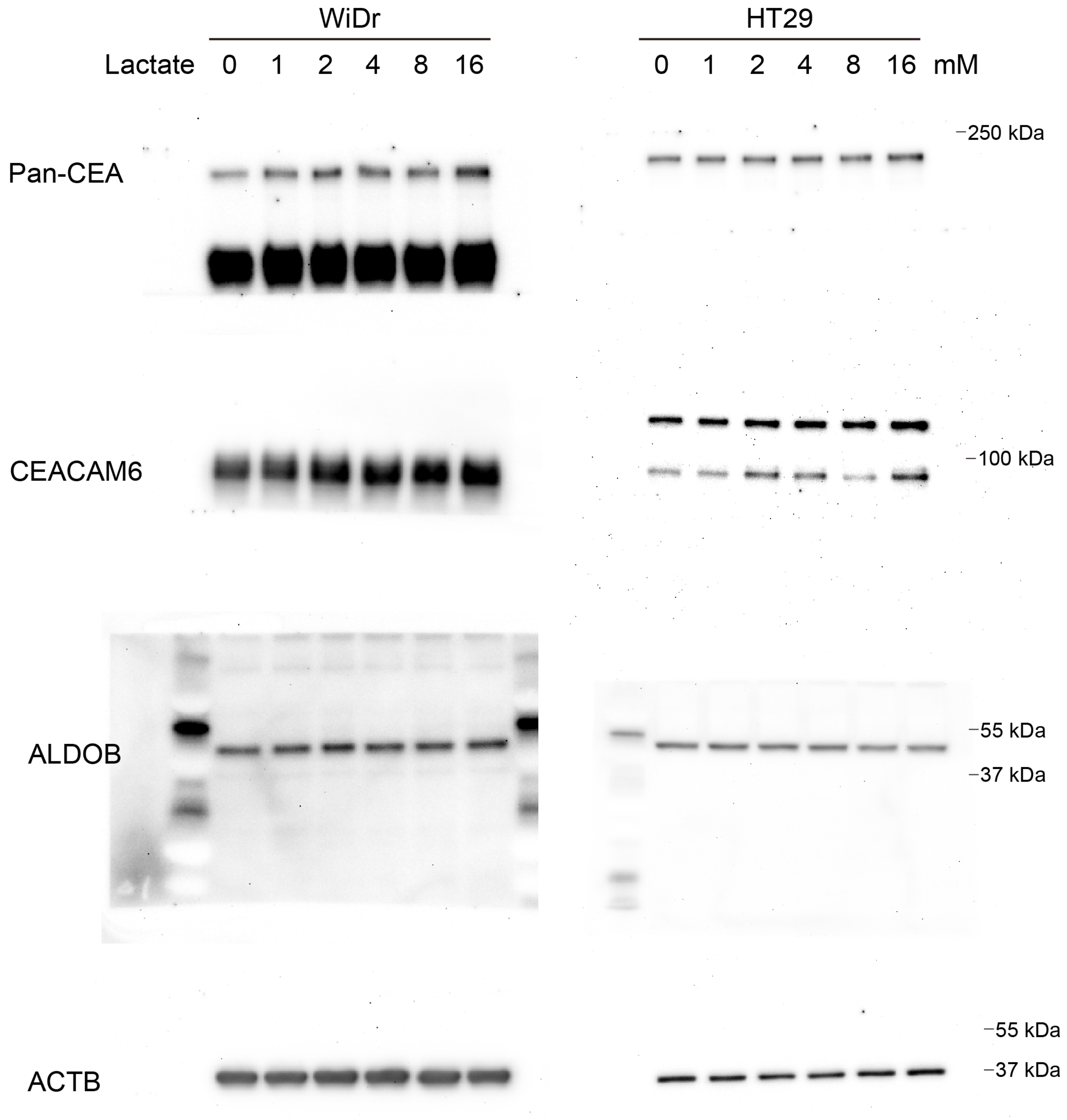

Raw image for Figure 4E

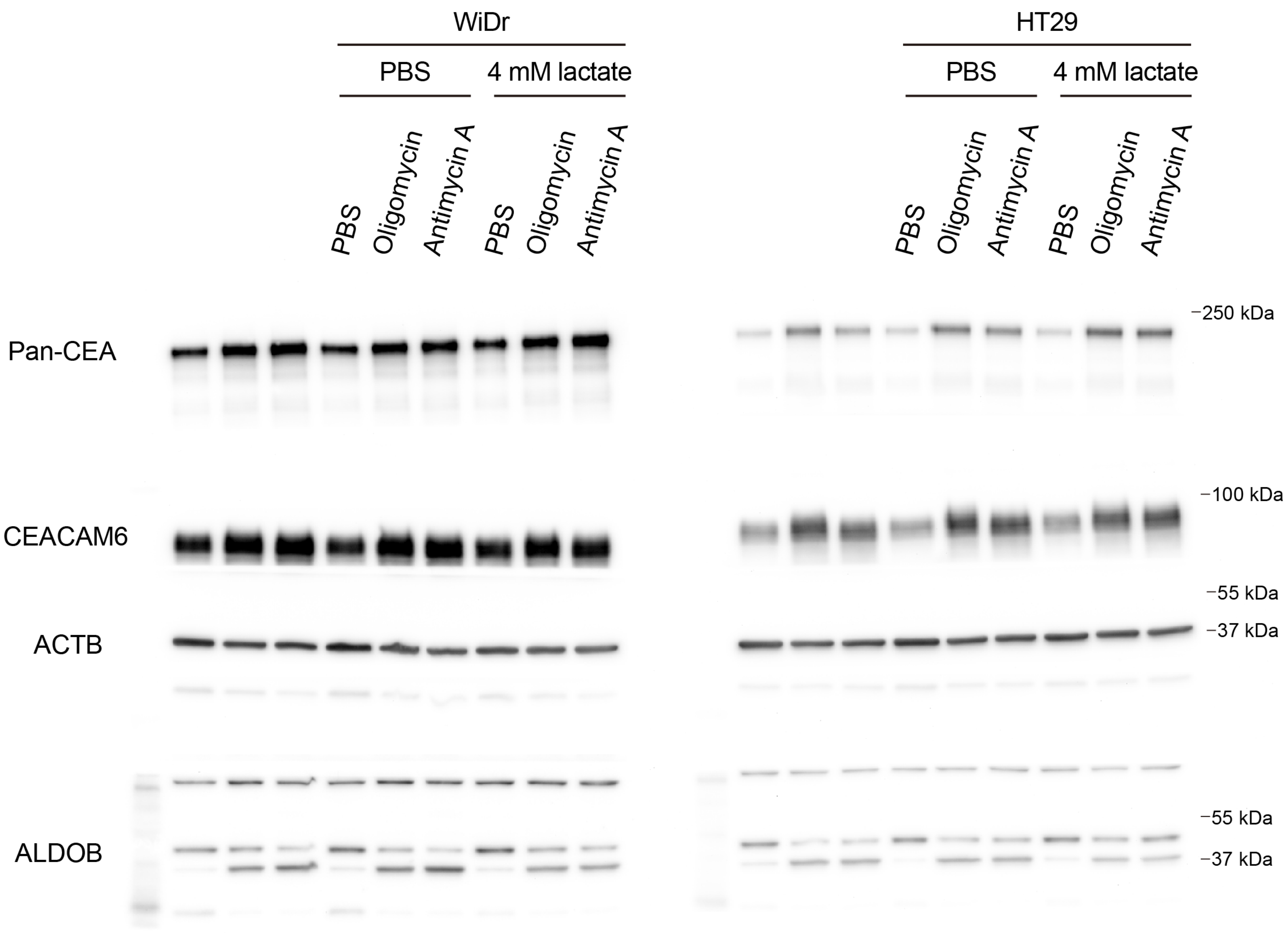

Raw image for Figure 4F

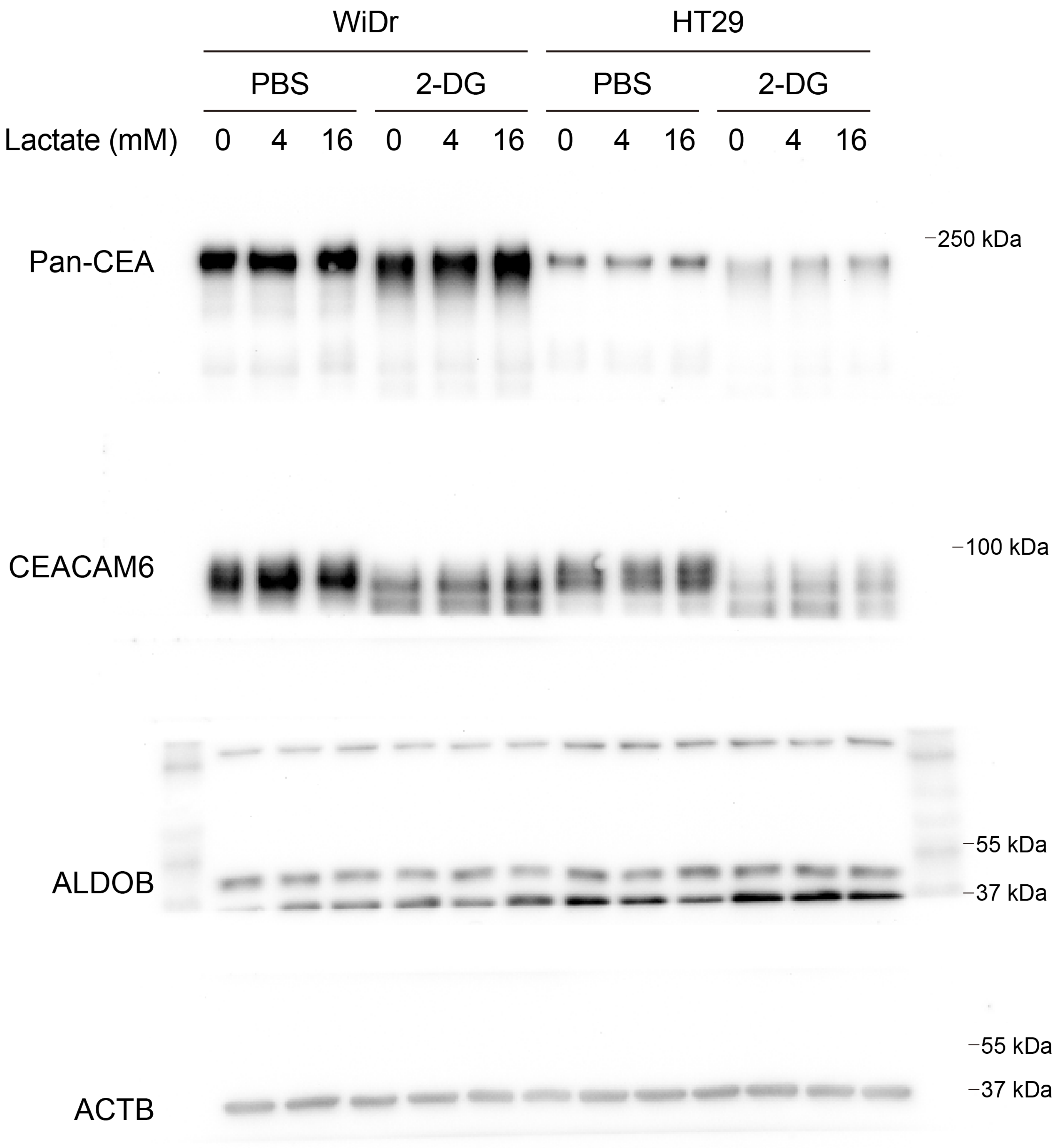

Raw image for Figure 5A

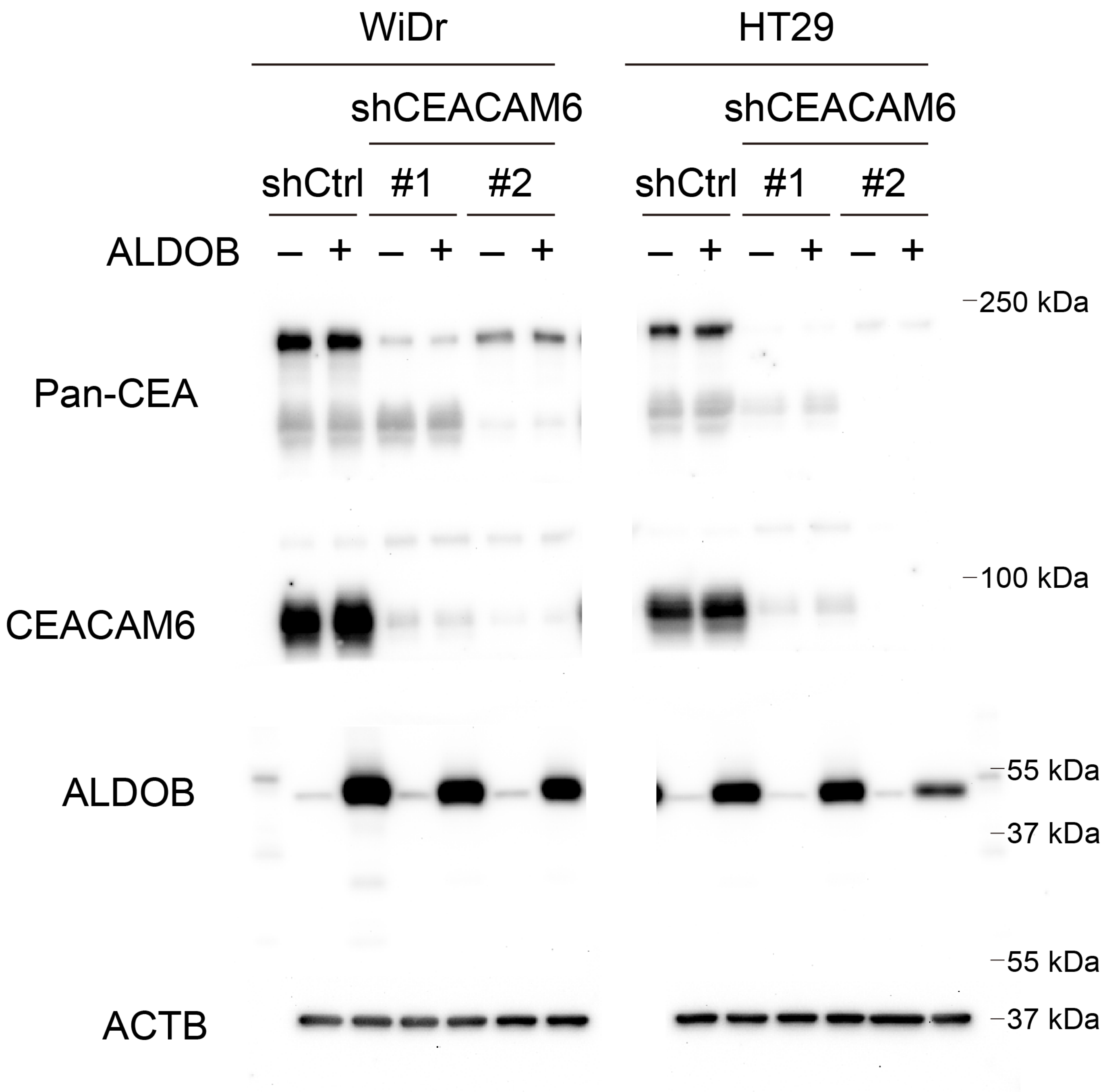

Raw image for Figure 5E

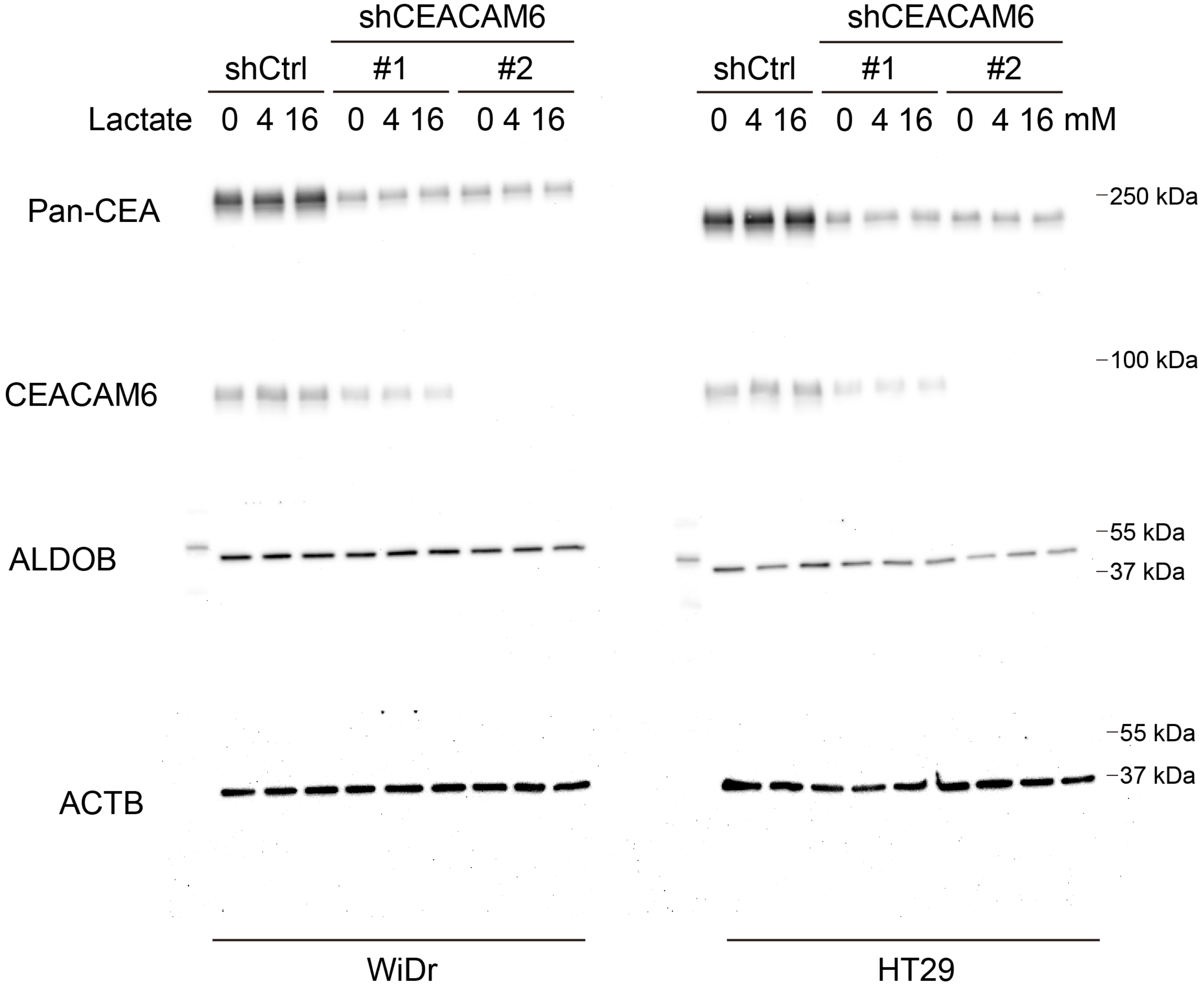

Raw image for Figure 6C

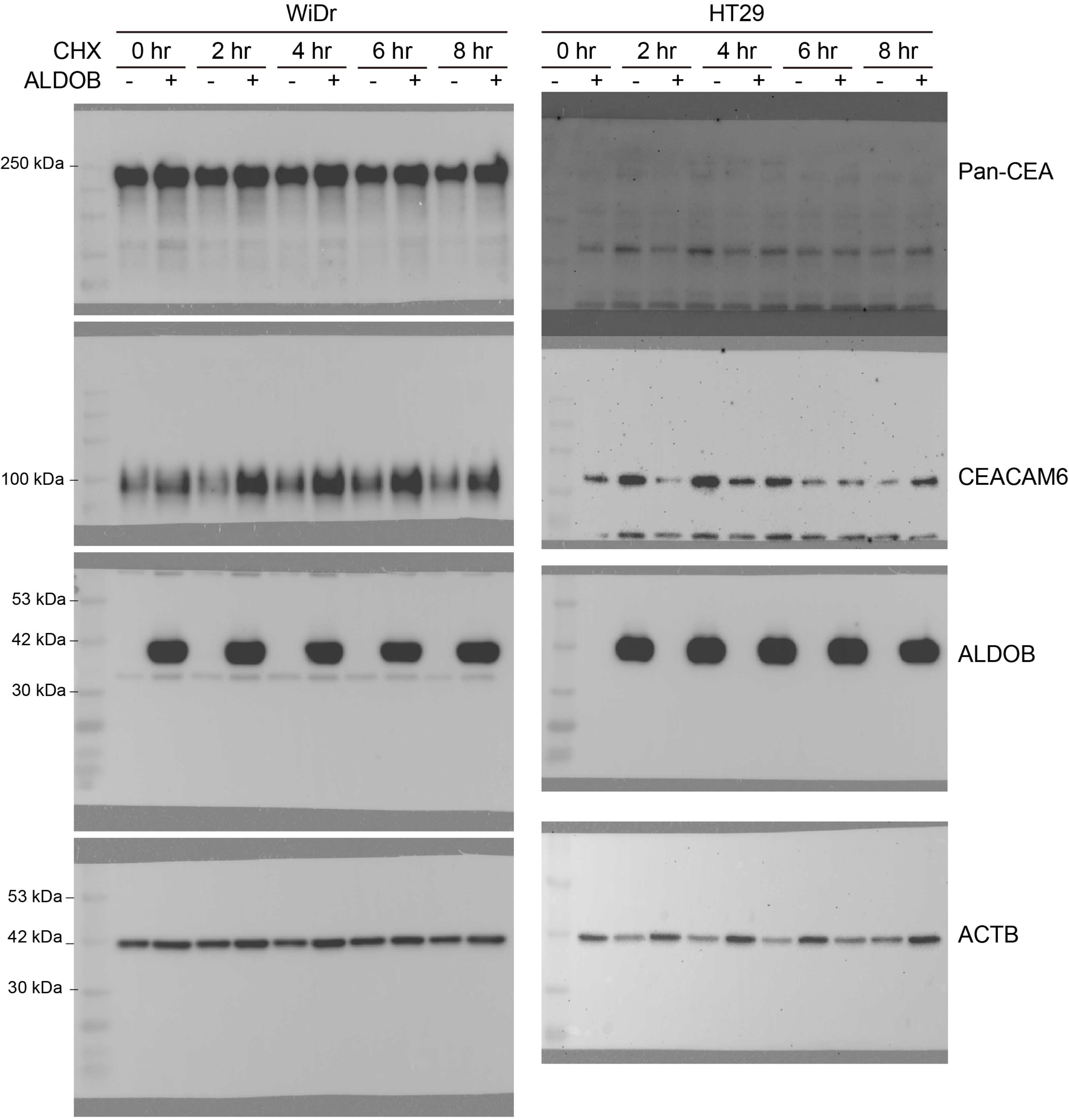

Raw image for Figure 6D

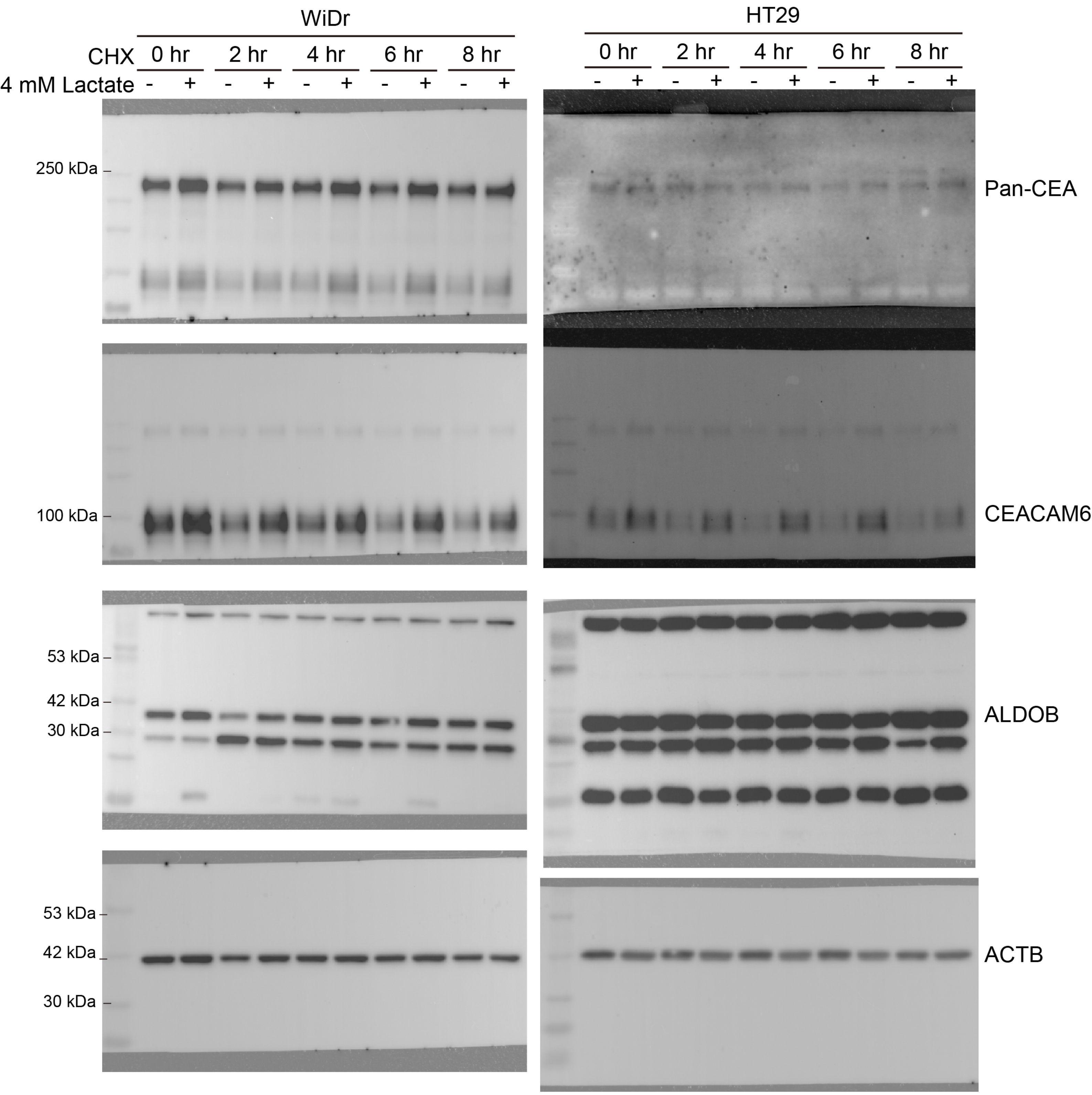

Raw image for Figure 6E

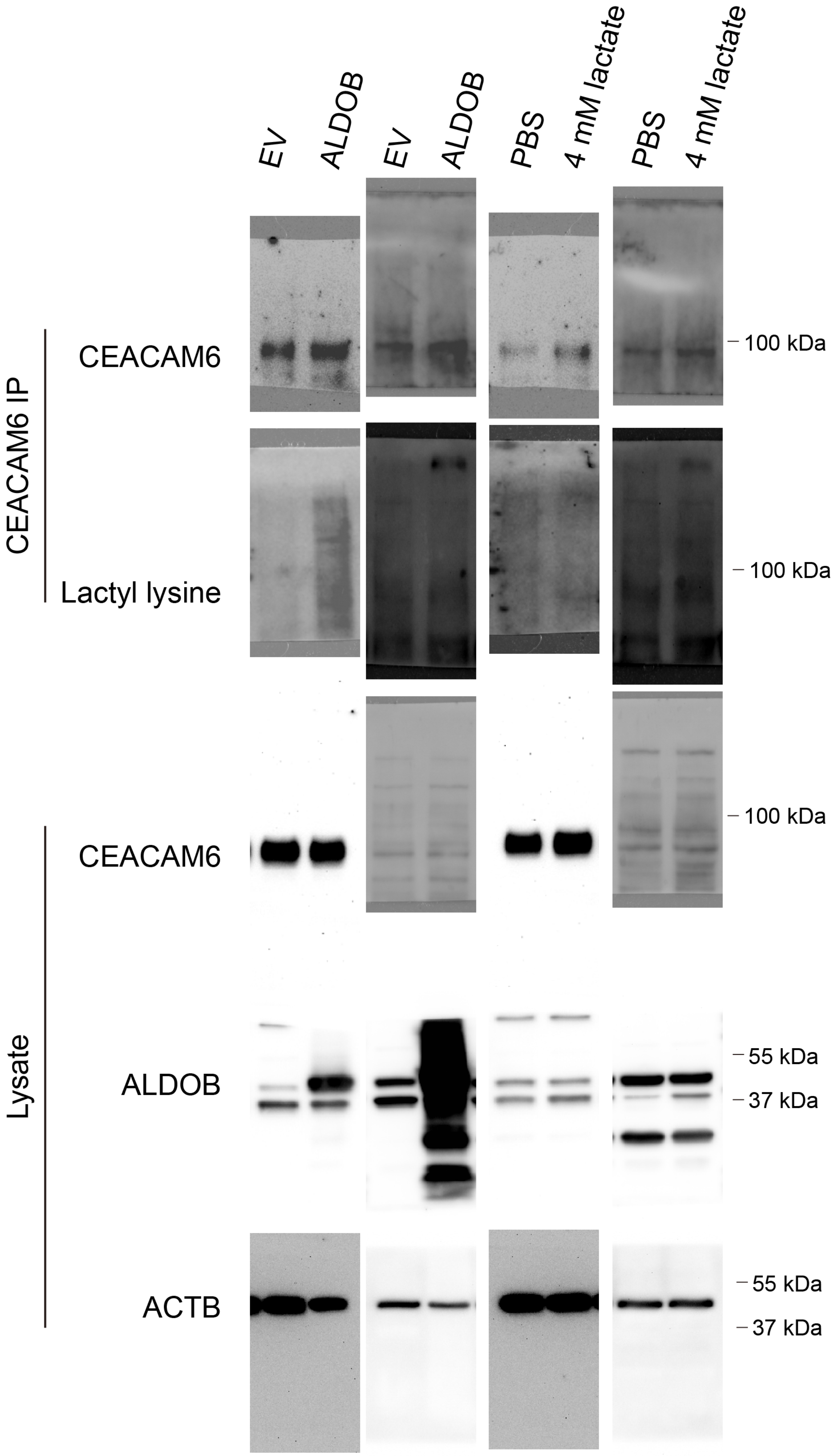

Supplement: Supplementary file 2 — Original Data File [file 41419_2023_6187_MOESM2_ESM.pdf]
