# Supplementary material for: Aldolase B-driven lactagenesis and CEACAM6 activation promote cell renewal and chemoresistance in colorectal cancer through the Warburg effect
Source: Cell Death Dis. 2023 Oct 10;14(10):660. doi: 10.1038/s41419-023-06187-z (PMC10564793; doi:10.1038/s41419-023-06187-z)

**Supplementary information**

**Supplementary Figures**


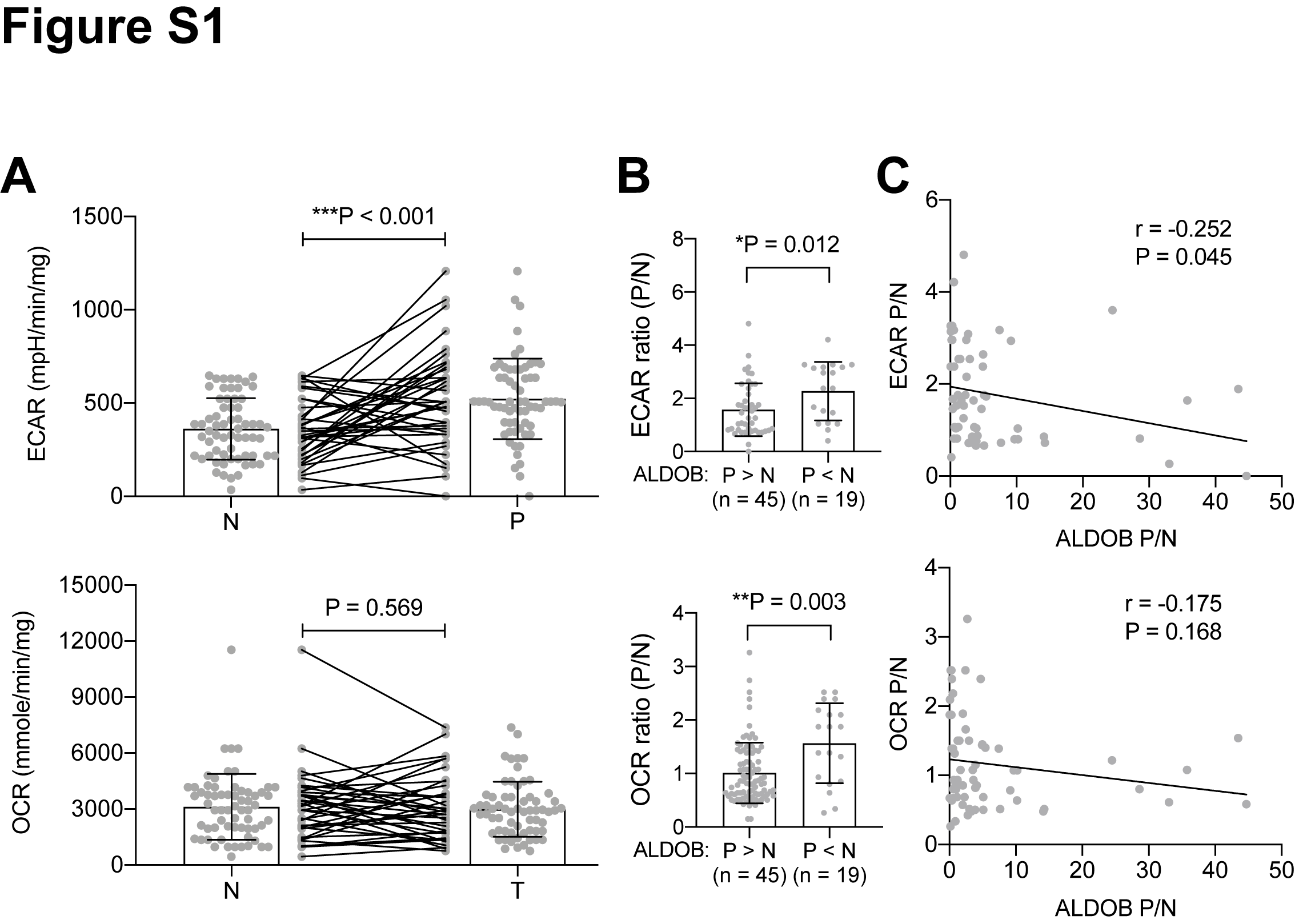


**Figure S1.** ALDOB expression is not associated with increased glycolysis in adenomatous polyp. (A) Extracellular acidification rate (ECAR) and oxygen consumption rate (OCR) obtained using the Seahorse assay. (B) Statistical analysis of bioenergetic changes in adenomatous polyp patients with high or low tumor ALDOB expression. (C) Pearson’s analysis of the correlation between ALDOB expression and bioenergetic changes in adenomatous polyp.


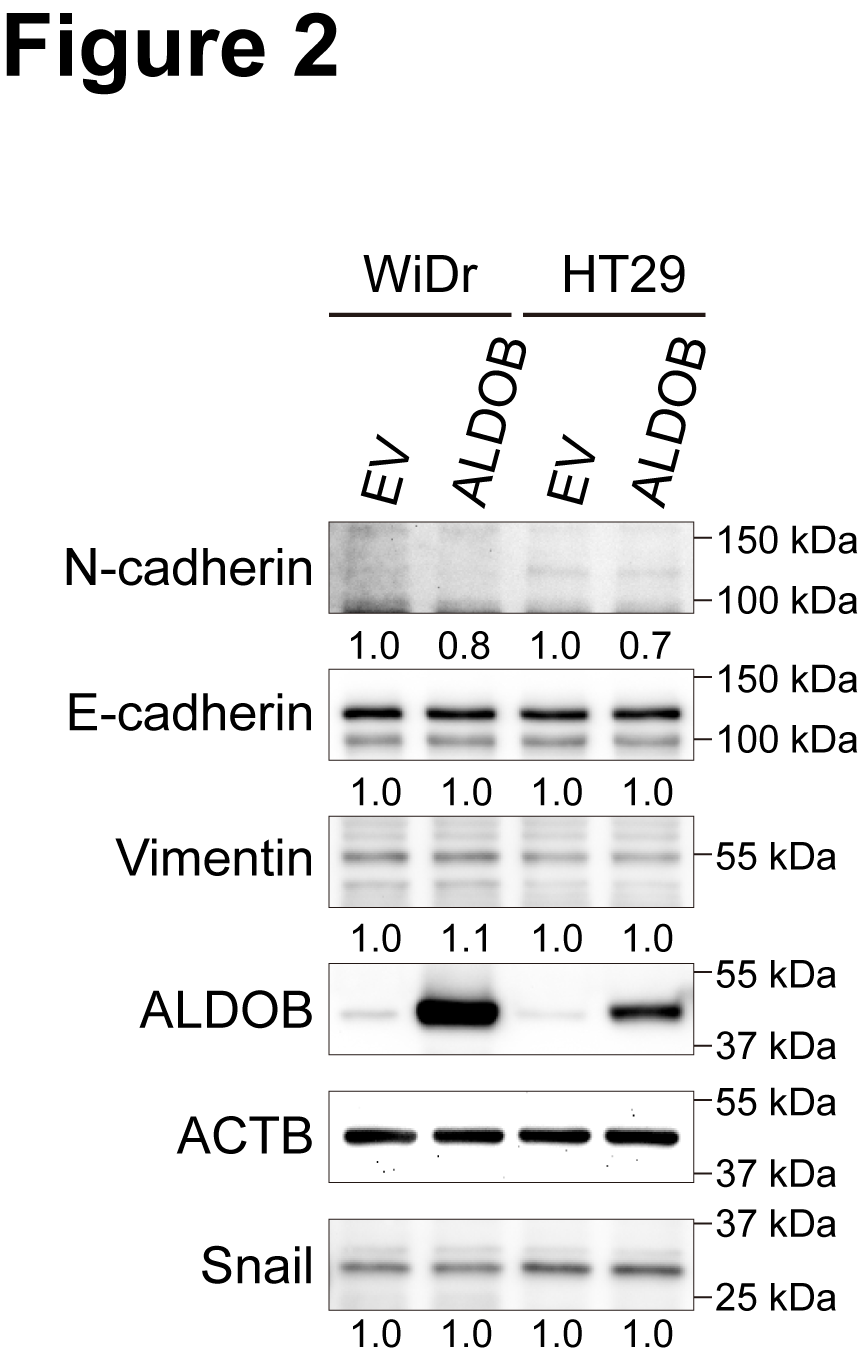


**Figure S2.** ALDOB does not promote epithelial-mesenchymal transition in WiDr and HT29 colorectal cancer cell lines. Representative Western blots showing the indicated protein levels in WiDr and HT29 colorectal cancer cells with and without ALDOB overexpression.


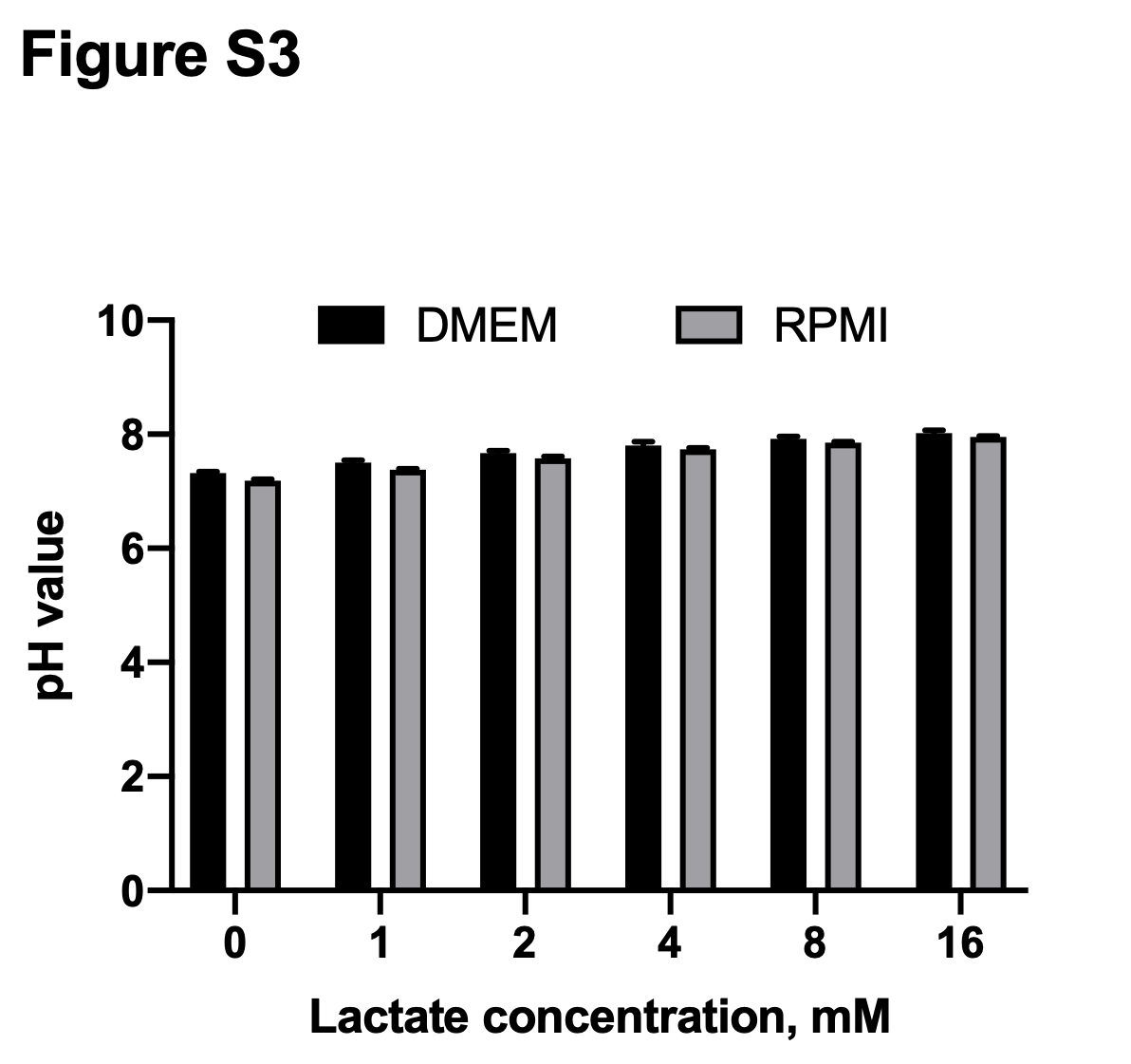


**Figure S3.** Impacts of different concentration of lactate on medium pH value change. The pH values of indicated medium were measured using a pH meter for three times.


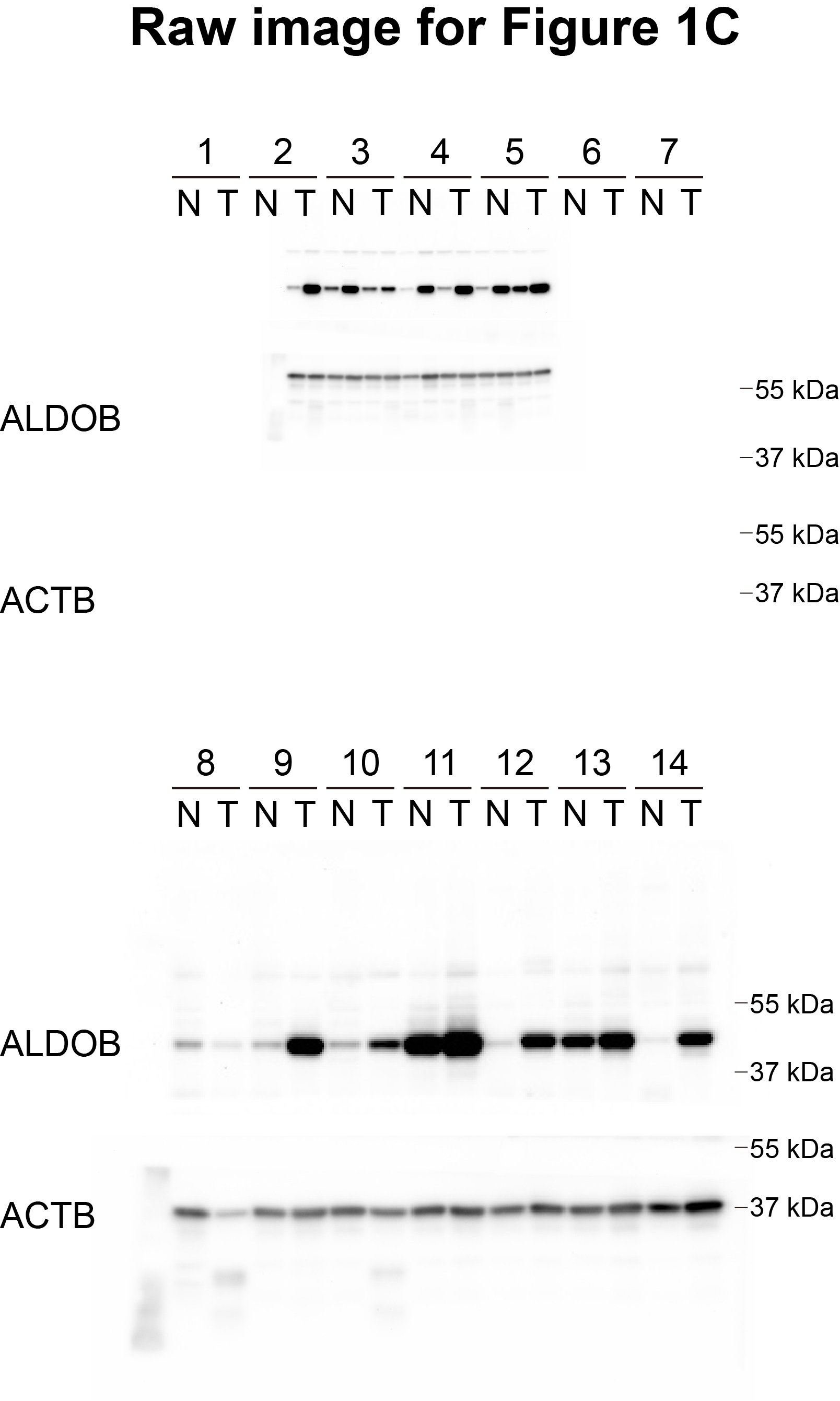


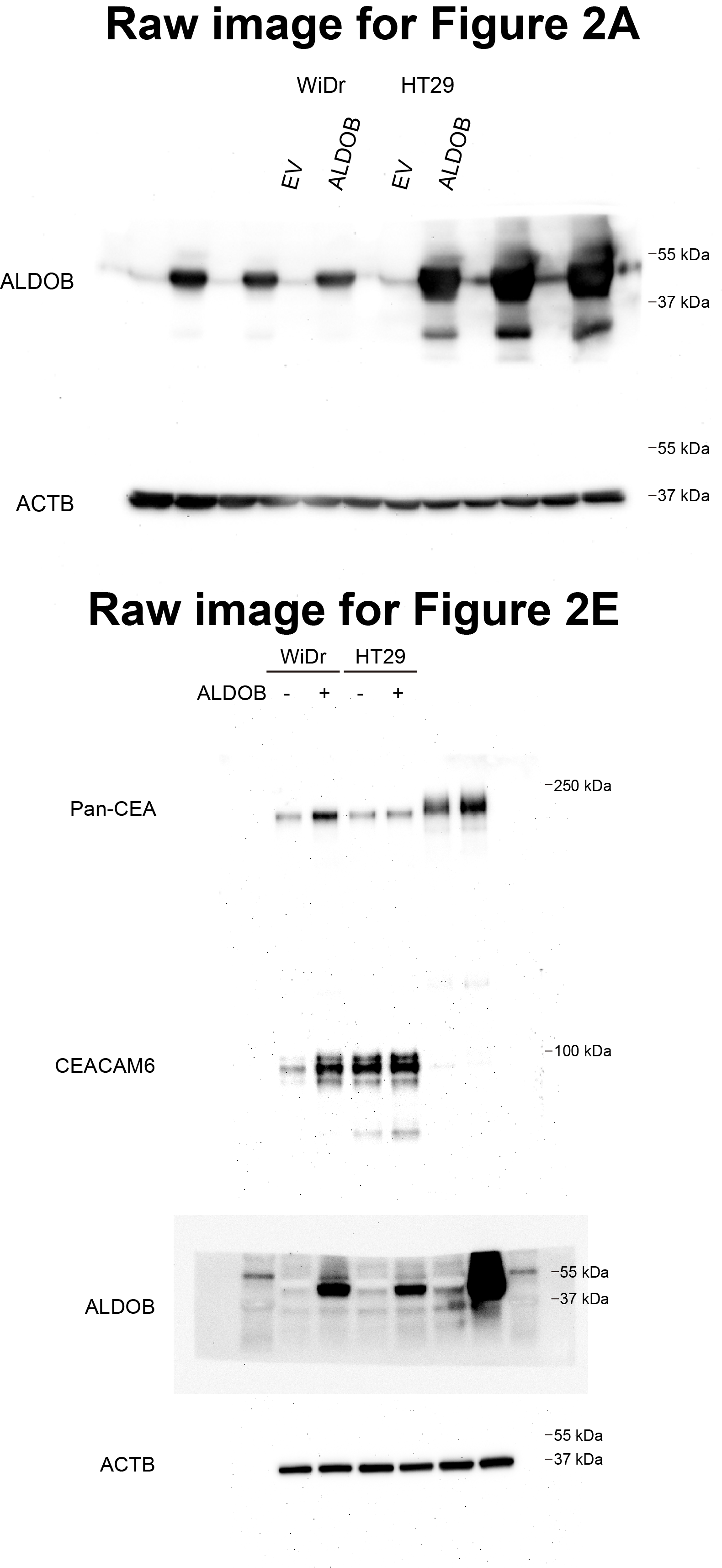


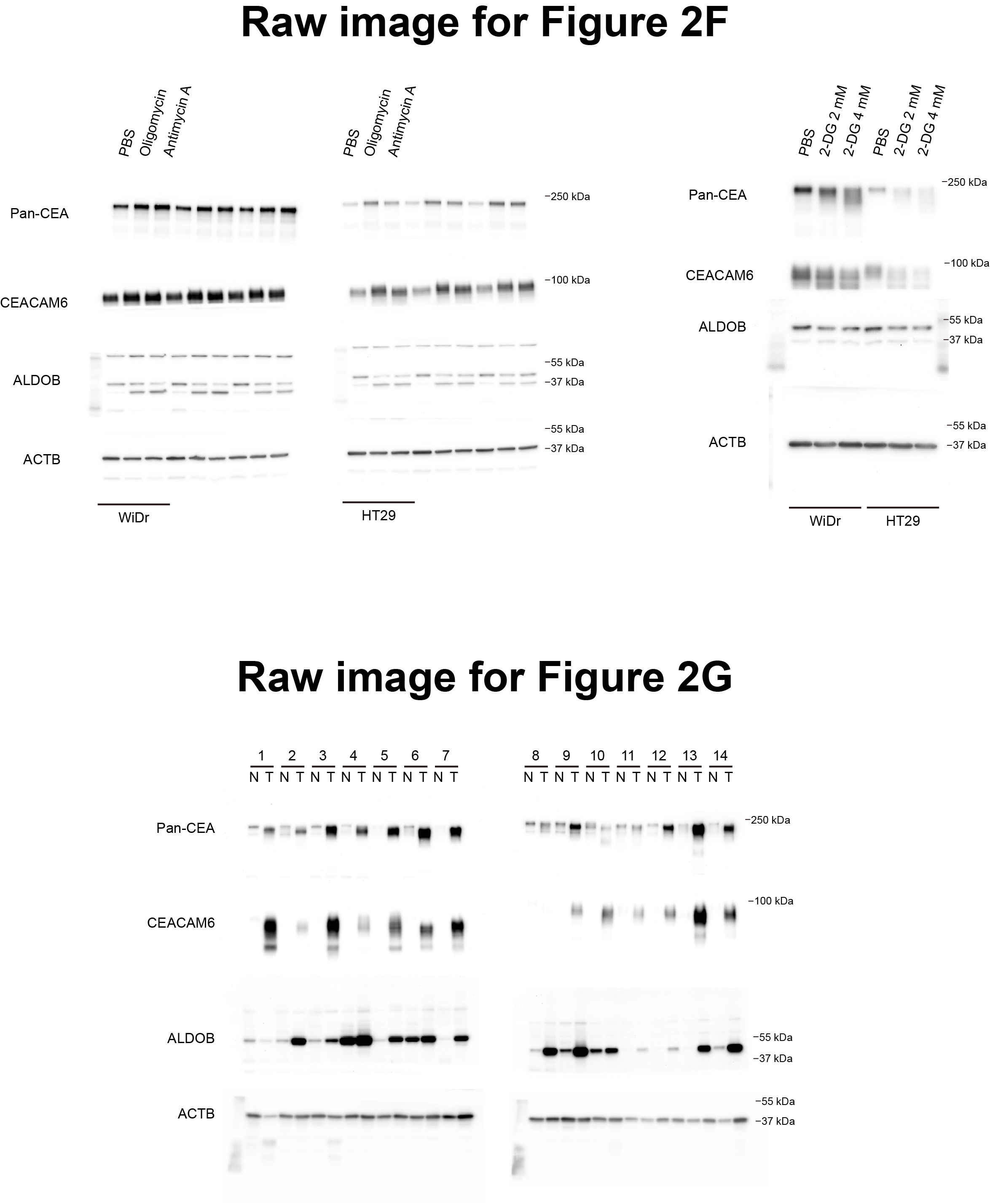


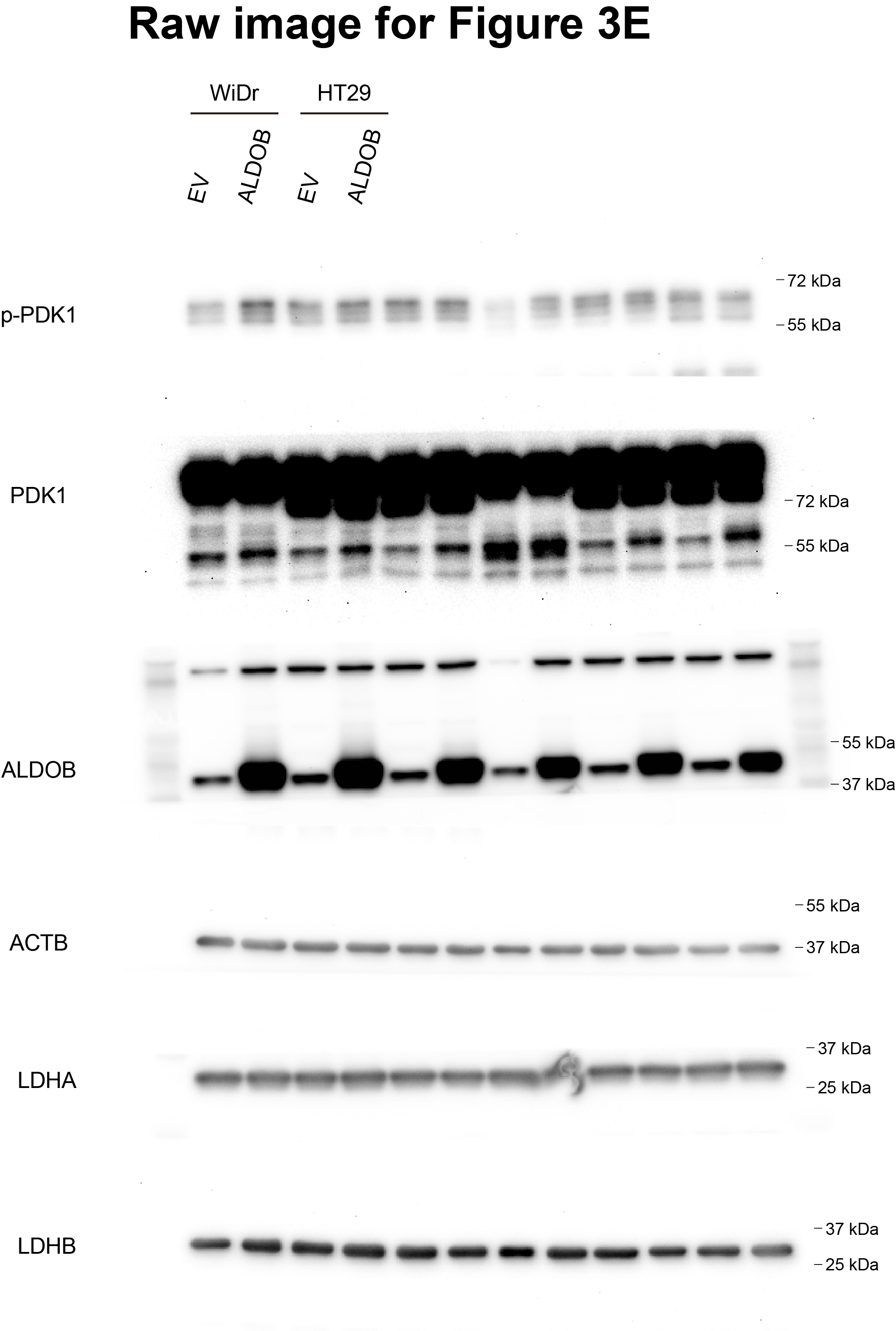


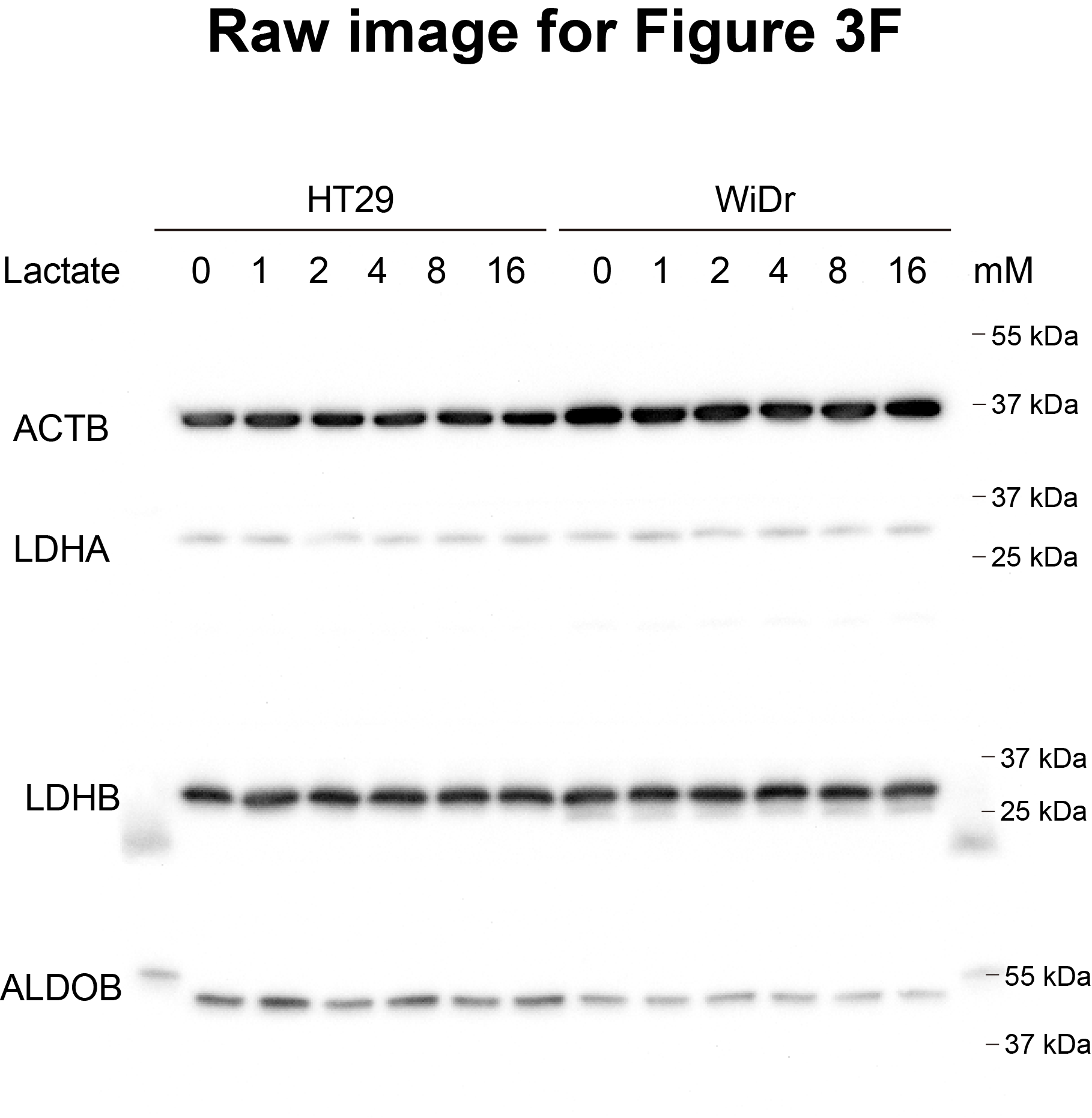


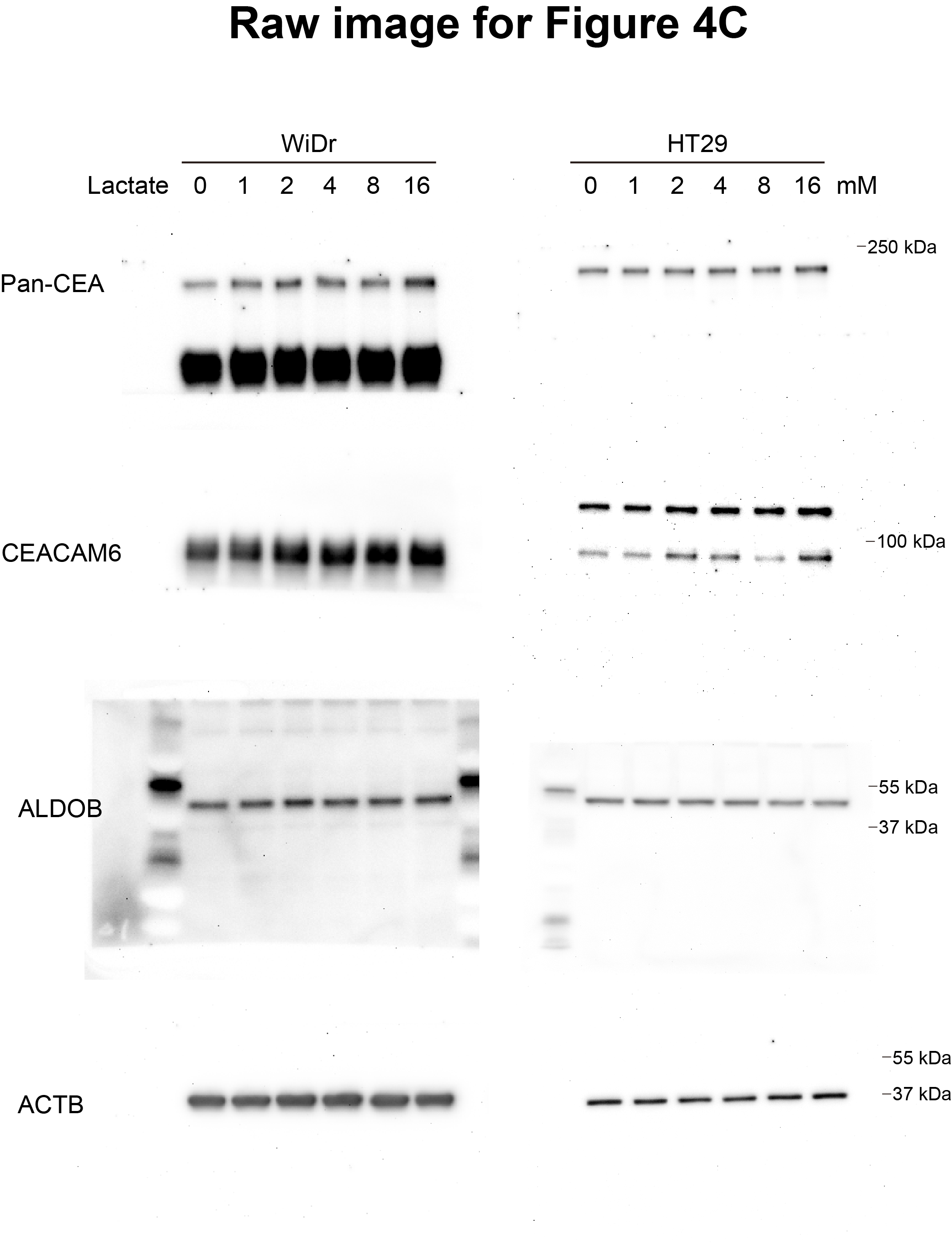


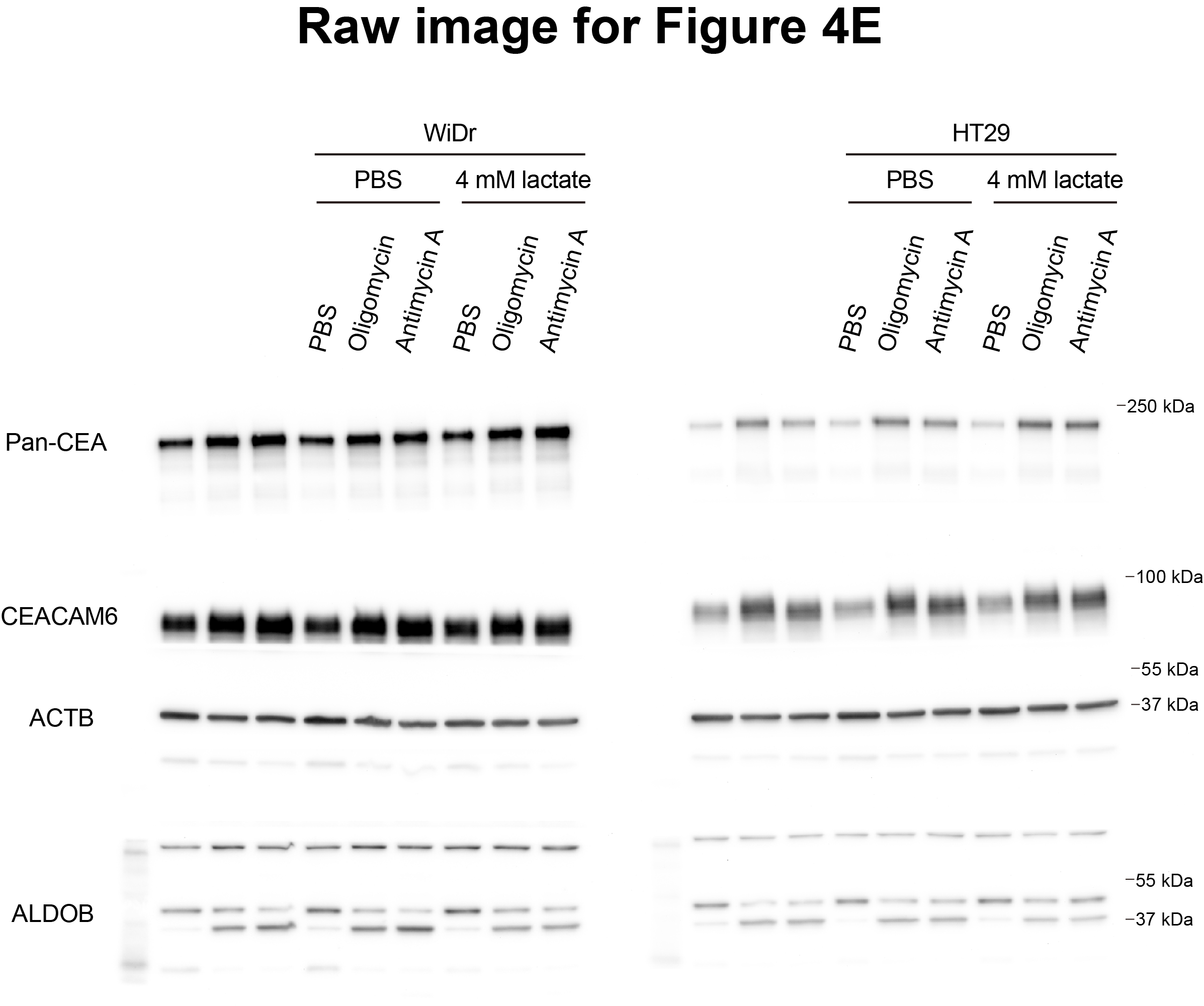


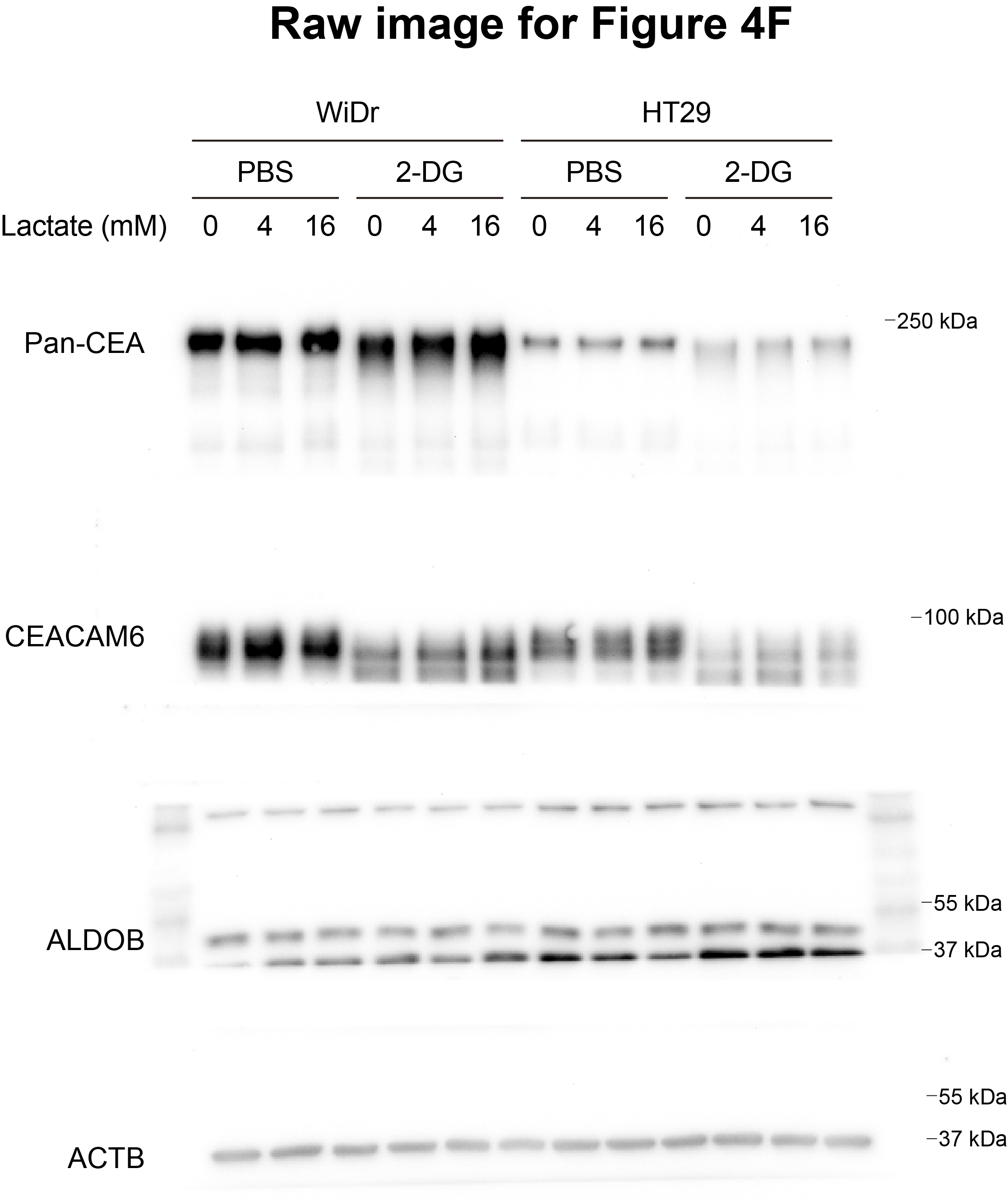


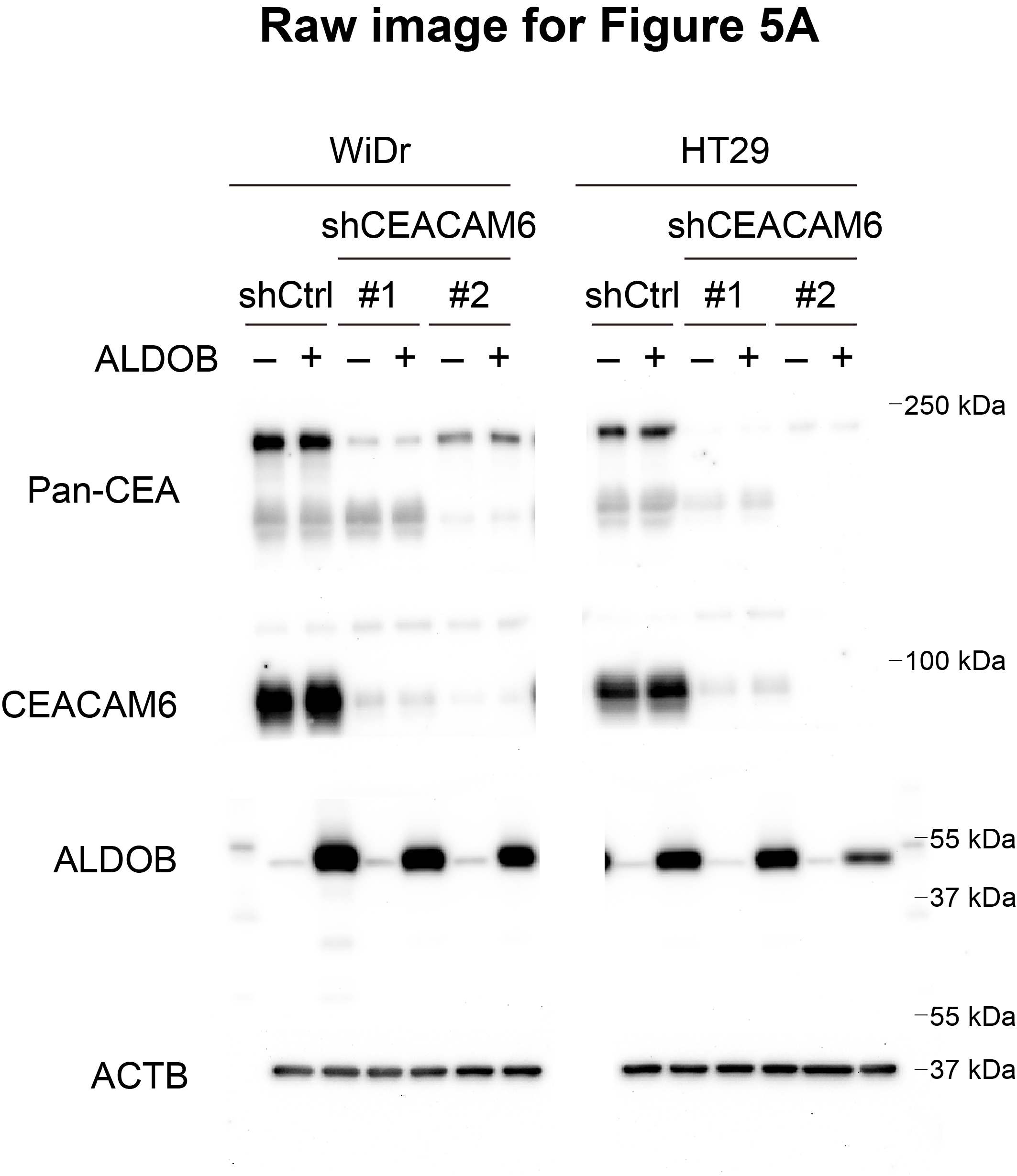


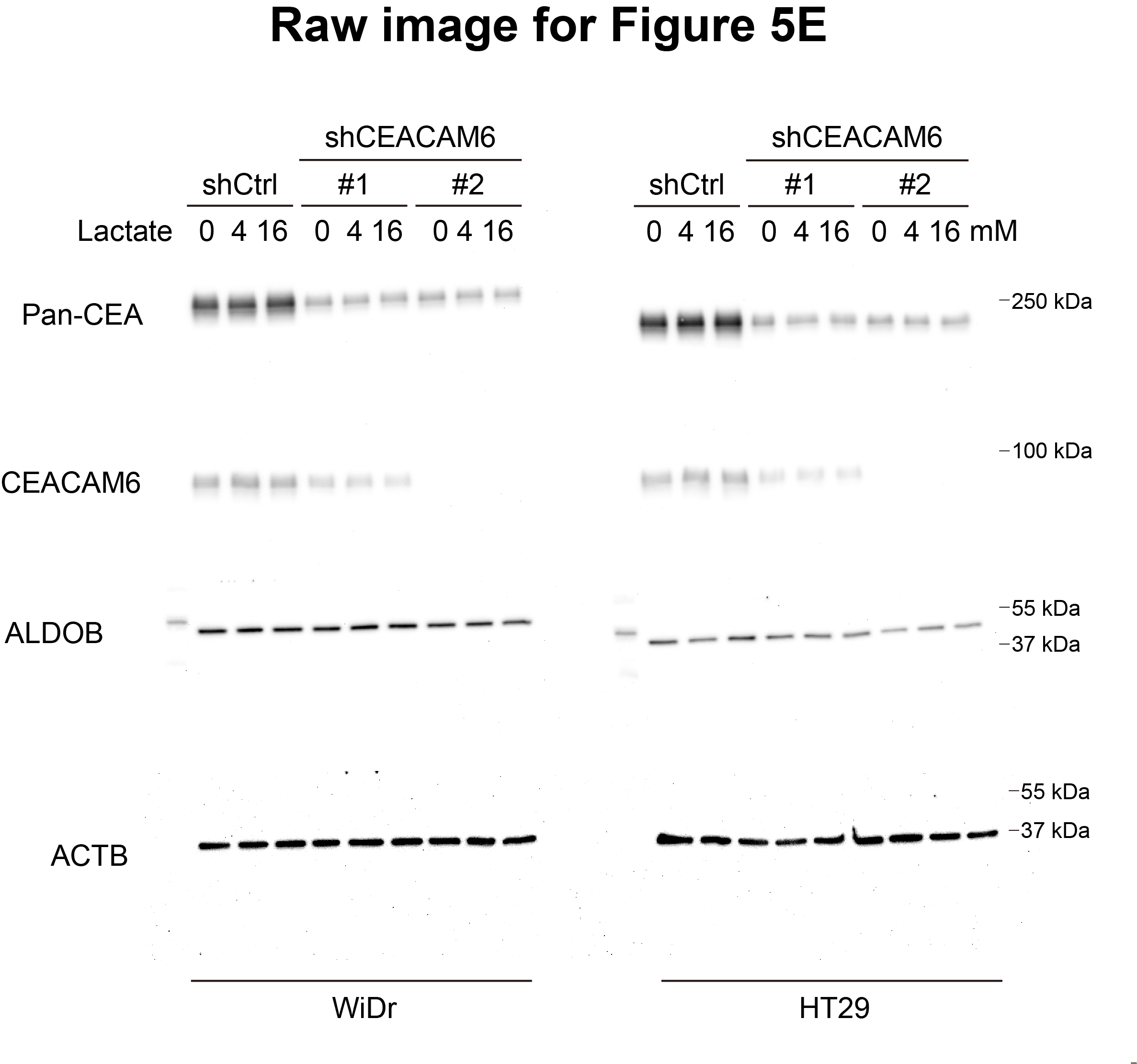

Supplement: Supplementary file 3 — Supplemental material [file 41419_2023_6187_MOESM3_ESM.docx]
